# Analytical approaches for evaluating passive acoustic monitoring data: A case study of avian vocalizations

**DOI:** 10.1002/ece3.8797

**Published:** 2022-04-21

**Authors:** Laurel B. Symes, Kyle D. Kittelberger, Sophia M. Stone, Richard T. Holmes, Jessica S. Jones, Itzel P. Castaneda Ruvalcaba, Michael S. Webster, Matthew P. Ayres

**Affiliations:** ^1^ 5922 K. Lisa Yang Center for Conservation Bioacoustics Cornell Lab of Ornithology Cornell University Ithaca New York USA; ^2^ 3728 Department of Biological Sciences Dartmouth College Hanover New Hampshire USA; ^3^ 56292 Smithsonian Tropical Research Institute Panama City Republic of Panama; ^4^ 3728 School of Biological Sciences University of Utah Salt Lake City Utah USA; ^5^ 5922 Macaulay Library Cornell Lab of Ornithology Cornell University Ithaca New York USA

**Keywords:** bioacoustics, biodiversity assessment, birdsong, Hubbard Brook Experimental Forest, passive acoustic monitoring, rarefaction

## Abstract

1. The interface between field biology and technology is energizing the collection of vast quantities of environmental data. Passive acoustic monitoring, the use of unattended recording devices to capture environmental sound, is an example where technological advances have facilitated an influx of data that routinely exceeds the capacity for analysis. Computational advances, particularly the integration of machine learning approaches, will support data extraction efforts. However, the analysis and interpretation of these data will require parallel growth in conceptual and technical approaches for data analysis. Here, we use a large hand‐annotated dataset to showcase analysis approaches that will become increasingly useful as datasets grow and data extraction can be partially automated.
2. We propose and demonstrate seven technical approaches for analyzing bioacoustic data. These include the following: (1) generating species lists and descriptions of vocal variation, (2) assessing how abiotic factors (e.g., rain and wind) impact vocalization rates, (3) testing for differences in community vocalization activity across sites and habitat types, (4) quantifying the phenology of vocal activity, (5) testing for spatiotemporal correlations in vocalizations within species, (6) among species, and (7) using rarefaction analysis to quantify diversity and optimize bioacoustic sampling.
3. To demonstrate these approaches, we sampled in 2016 and 2018 and used hand annotations of 129,866 bird vocalizations from two forests in New Hampshire, USA, including sites in the Hubbard Brook Experiment Forest where bioacoustic data could be integrated with more than 50 years of observer‐based avian studies. Acoustic monitoring revealed differences in community patterns in vocalization activity between forests of different ages, as well as between nearby similar watersheds. Of numerous environmental variables that were evaluated, background noise was most clearly related to vocalization rates. The songbird community included one cluster of species where vocalization rates declined as ambient noise increased and another cluster where vocalization rates declined over the nesting season. In some common species, the number of vocalizations produced per day was correlated at scales of up to 15 km. Rarefaction analyses showed that adding sampling sites increased species detections more than adding sampling days.
4. Although our analyses used hand‐annotated data, the methods will extend readily to large‐scale automated detection of vocalization events. Such data are likely to become increasingly available as autonomous recording units become more advanced, affordable, and power efficient. Passive acoustic monitoring with human or automated identification at the species level offers growing potential to complement observer‐based studies of avian ecology.

## Graphical abstract

Relationship between total number of bird species detected via passive acoustic monitoring and the number of recorder days that were analyzed, as measured at Hubbard Brook Forest in New Hampshire.

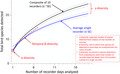

## 1 INTRODUCTION

Ecological insights and informed conservation rely on understanding when and where organisms occur (Fisher et al., 1943; MacArthur, 1984). Ecologists and conservation biologists have used many different approaches to document the distribution of organisms, ranging from detailed observations by skilled field personnel to aerial overflights and analysis of trace environmental DNA (Dejong & Emlen, 1985; Ficetola et al., 2008; Hodgson et al., 2016; Scott et al., 1981). Technological advances continue to provide new avenues for monitoring habitats, with acoustic analysis rapidly gaining prominence as a powerful method for assessing the distribution and behavior of animals (Sugai et al., 2019; Wood et al., 2019).

Passive acoustic monitoring (PAM) is a sampling approach that uses unattended audio recorders to sample sounds over large swaths of space and time (Sugai et al., 2019). Autonomous recording units (ARUs) collect data without the presence of a human observer and provide an enduring record of habitat use, behavioral patterns, phenology, and changes in sound production by wildlife over time (Davis et al., 2017; Desjonquères et al., 2020; Wood et al., 2019a). Passive acoustic monitoring also facilitates the detection of species that are uncommon, secretive, or occur during seasons, times of day, or weather conditions when human observers are less likely to sample (Sebastián‐González et al., 2018). As autonomous recording units become more advanced, affordable, and power efficient, passive acoustic monitoring offers a complementary and non‐invasive approach for ecological studies and biodiversity monitoring (Gibb et al., 2019; Potamitis, 2014; Sebastián‐González et al., 2018; Sugai et al., 2019; Xie et al., 2018). Furthermore, automated ARUs allow for broader temporal and spatial sampling and minimize the potential for in‐field observer bias (Sugai et al., 2019).

Currently, passive acoustic monitoring data are often analyzed by humans who review spectrograms (visual images of acoustic information) and listen to audio recordings to identify species. However, manual annotation is time‐consuming and limits the amount of data that can be processed. The ability to survey many more locations for longer periods of time provides crucial data, but also raises new challenges and opportunities in data analysis. Advancements in automation, particularly machine learning approaches, are poised to accelerate and scale annotation dramatically (Kahl et al., 2018; Shiu et al., 2020; Vickers et al., 2019). Advances in data extraction capacity must therefore be met by parallel advancements in methodological frameworks and statistical analysis (Gasc et al., 2017; Gibb et al., 2019; Sebastián‐González et al., 2018; Wood et al., 2021).

Much work has been done on the marine soundscape, with a long and robust history of using acoustics to study marine mammals (Lin et al., 2016; Marques et al., 2009, 2011; Matthews et al., 2014; Rice et al., 2019). However, the application of passive acoustic monitoring to terrestrial systems is more recent (Sebastián‐González et al., 2018; Sugai et al., 2019), with studies utilizing passive acoustic monitoring becoming more widespread in the mid‐2000s (Sugai et al., 2019). Although some of the approaches that we consider may be relevant to marine work, our focus here is on soundscape approaches for terrestrial animals that vocalize frequently, such as birds, anurans, and some mammals. Compared to other terrestrial organisms, birds are one of the best‐known and best‐studied taxonomic groups, with vocalizations that are used in diverse contexts, including territoriality and resource defense, attraction of mates, and alerting other birds to the presence of a predator (Webster & Podos, 2018).

We develop and present methods for the refinement and analysis of acoustic data obtained from passive acoustic monitoring. We begin with methods that are currently used with small manually generated datasets but are suitable for expansion to much larger datasets. We then present analytical approaches that are only feasible with large samples in space and time. These approaches include: (1) the generation of species lists (Lellouch et al., 2014; Luther, 2009) and descriptions of vocal variation in traits such as duration and frequency (e.g., Duan et al., 2011; Planqué & Slabbekoorn, 2008; Potamitis, 2014; Towsey et al., 2012; Xie et al., 2018), providing an account of the species detected in a given area and time period. If vocalization rates are measured on a fine‐grained scale (e.g., minutes, hours, or days), it becomes possible to estimate, (2) how vocalization rates are impacted by abiotic factors such as precipitation (Bruni et al., 2014; Hasan, 2010; Keast, 1994; Lengagne & Slater, 2002), wind (Hasan, 2010; Lengagne et al., 1999), and temperature (Bruni et al., 2014; Gottlander, 1987; Keast, 1994; Thomas, 1999). Passive acoustic data can also be combined with information about habitat type and land‐use history to produce (3) community patterns in vocalization activity across sites (Depraetere et al., 2012; Gasc et al., 2013; Rodriguez et al., 2014). Detailed data on vocalizations over time also make it possible to quantify the (4) timing of vocal activity, such as changes in acoustic signaling across hours to days (Gasc et al., 2013; Rodriguez et al., 2014; Towsey et al., 2012) or months to years (Towsey et al., 2014). This approach can be used directly to answer research questions, such as whether a warming climate shifts activity dates (Llusia et al., 2013), or to control for the impact of phenology and diurnal patterns on other analyses and comparisons. Large‐scale synchronized recording can also provide a novel tool for behavioral research (Tobias et al., 2014). By deploying passive acoustic recorders that record multiple locations simultaneously, it becomes possible to apply statistical tools from population ecology to test for (5) spatiotemporal correlations in vocalizations within species and (6) among species (e.g., Brumm, 2006; Burt & Vehrencamp, 2005; Laiolo et al., 2011; Luther, 2009; Planqué & Slabbekoorn, 2008; Tobias et al., 2014). Finally, (7) Species accumulation functions and optimization of bioacoustic sampling schemes use rarefaction analyses to describe species richness across scales and can aid in planning and designing acoustic sampling schemes (Dixon et al., 2020; Marín‐Gómez et al., 2020; Naithani et al., 2018). To demonstrate these approaches, we used passive acoustic recordings of the dawn birdsong chorus, with manual counts of the number of vocalizations per species per unit time. These approaches provide a package of tools for approaching and interpreting acoustic data to test ecological hypotheses and assess biodiversity across space and time.

## 2 METHODS

### 2.1 Study sites

**FIGURE 1:**
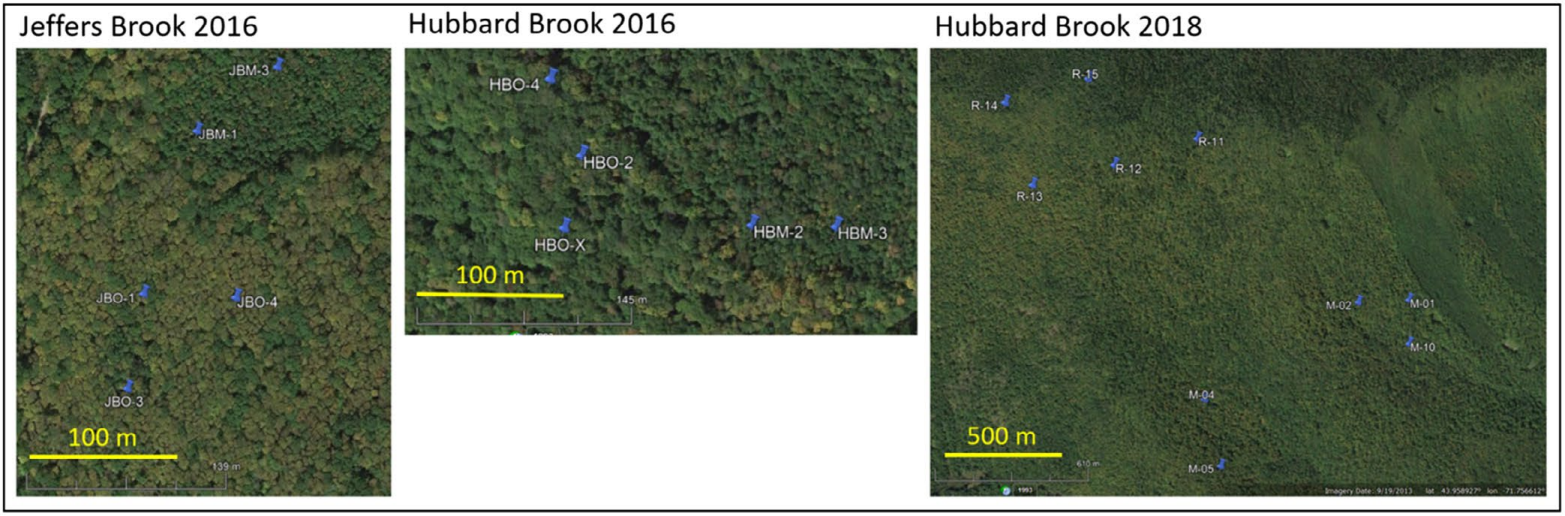
Relative positions of audio recorders within study sites in Jeffers Brook and Hubbard Brook watersheds, NH. Coordinates and site characteristics are in Table 1. Base images are from Google Earth

Acoustic sampling was conducted in hardwood forests at 500–800 m elevation in the White Mountains of New Hampshire, USA. Study sites (Figure 1, Table S1) were located in the Hubbard Brook Experimental Forest and in a similar habitat within the Jeffers Brook Forest, approximately 15 km from Hubbard Brook (Table S1). Hubbard Brook Experimental Forest was established in 1955 with a focus on hydrologic and forest science (Holmes & Likens, 2016), and studies of avian ecology have been running continuously since 1969 (e.g., Holmes, 2011; Holmes et al., 1979; Holmes & Sherry, 2001; Holmes & Sturges, 1975; Townsend et al., 2013). The forests at both Hubbard Brook and Jeffers Brook consist of variably aged second growth northern hardwoods, dominated by sugar maple (*Acer saccharum*), American beech (*Fagus grandifolia*) and yellow birch (*Betula alleghaniensis*), with occasional white ash (*Fraxinus americana*), white birch (*Betula papyrifera*), red spruce (*Picea rubens*), and balsam fir (*Abies balsamea*) (Campbell et al., 2007). The understory included saplings of the canopy species (especially American beech) as well as patches of hobblebush (*Viburnum lantanoides*) and occasional striped maple (*Acer pensylvanicum*). Both sites contained relatively mature forests (last harvested in 1910–1915) and middle‐aged stands (clearcut in 1970–1975) (Goswami et al., 2018). In mid‐aged stands compared to mature stands, the diameters of the largest trees were smaller (<40 versus up to ~50 cm diameter), but trees per hectare was higher with the result that above‐ground biomass was similar (basal area = 25–35 m^2^/ha). Bird vocalizations from the two ages of forests (hereafter “mature” and “mid‐aged,” respectively) were sampled at both sites to compare acoustic samples from nearby forests of different successional stages.

### 2.2 Data collection and recording hardware

This paper contains two acoustic datasets, one collected in 2016 and the other in 2018. In 2016, we conducted sampling to compare avian vocalizations in mid‐aged and mature forest stands, replicated across Hubbard Brook and Jeffers Brook watersheds. In 2018, sampling was concentrated in Hubbard Brook forest and designed to provide high resolution within one forest area, allowing for more detailed examination of spatial patterning in vocalization activity. In both years, recorders were activated each morning for a 10‐minute period spanning 06:20–06:30 local time (UTC‐4). Depending on the date, the recordings started at 55–75 min after sunrise. The 10‐min interval for recording bouts parallels a common point count duration (Buskirk & McDonald, 1995) and was chosen to be long enough to capture most species vocalizing at that site on that morning, but short enough that we could still annotate many different mornings and compare inter‐ and intraspecific patterning of vocalizations among days (Tobias et al., 2014). For annotation, we chose a sample size of 20 dates per year as being both sufficient and manageable. We selected the dates within years such that they were distributed throughout the period of available recordings, but with longer intervals between dates later in the season when there were generally fewer vocalizations. Dates for annotation were chosen in advance of examining the sound recordings and so were not biased with respect to vocalization activity or ambient sound levels.

We used Olympus DS‐40 recorders (Olympus, Center Valley, PA, USA) deployed in plastic boxes and connected to their original microphone by a 1‐m extender cable. Each microphone was placed at a height of 2 m and was suspended below a fabric rain shield 25 cm in diameter. The recorders generated MP3 files with a sampling rate of 44.1 kHz on the “high‐quality” setting, with the manufacturer's maximum microphone sensitivity, no frequency filter, and no variable control voice actuator. The MP3 files were converted to 16 bit WAV files using Switch Plus converter (NCH software, Canberra, Australia) so that recordings could be digitally analyzed and manipulated. The compressed MP3 format discards some high‐frequency information, resulting in smaller files, but lower acoustic resolution, particularly at frequencies higher than those used by most bird species. These missing data are not recovered with the conversion to WAV format.

In 2016, three recorders were in the mature forest and two were in mid‐aged forest in both the Hubbard and Jeffers Brook forests. Within watersheds, recorders were separated by 50–200 m, minimizing the chances that an individual songbird was captured by multiple recording devices. Recorders were activated for 10 min each morning from 09 June to 21 July. In 2016, the estimated dates of leaf out of canopy trees and median initiation of first clutch by a representative locally breeding bird, the Black‐throated Blue Warbler (*Setophaga caerulescens*), were approximately 12 May and 31 May, respectively (Lany et al., 2016). We analyzed audio data for the following 20 dates in 2016: 09‐Jun, 11‐Jun, 15‐Jun, 16‐Jun, 18‐Jun, 20‐Jun, 24‐Jun, 26‐Jun, 28‐Jun, 30‐Jun, 01‐Jul, 03‐Jul, 04‐Jul, 06‐Jul, 07‐Jul, 08‐Jul, 14‐Jul, 16‐Jul, 19‐Jul, and 21‐Jul.

In 2018, we sampled vocalizations only within Hubbard Brook Experimental Forest. The 10 recorders were distributed across an area that has been the focus of long‐term studies of breeding songbirds (Holmes et al., 1986; Holmes, 2011; Rodenhouse & Holmes, 1992; Townsend et al., 2013). Distances between recorders ranged from 176 to 1729 m (Figure 1), allowing us to test for correlations in behavior at different distances. Recorders were activated for 10 min each morning from 20 May to 15 August. The estimated dates of leaf‐out and median initiation of first clutch by Black‐throated Blue Warblers in 2018 were 10 May and 30 May. We analyzed audio data for the following 21 dates in 2018: 13‐May, 15‐May, 17‐May, 20‐May, 23‐May, 26‐May, 29‐May, 01‐Jun, 04‐Jun, 07‐Jun, 10‐Jun, 13‐Jun, 16‐Jun, 19‐Jun, 22‐Jun, 25‐Jun, 28‐Jun, 01‐Jul, 04‐Jul, 07‐Jul, 10‐Jul.

All 410 10‐minute recordings (200 from 2016 and 210 from 2018), and their associated metadata are available at Symes et al. (2021).

### 2.3 Data selection and annotation

Recordings were annotated with species names by listening to sound recordings and looking at spectrograms (visual and auditory review). To review recordings visually we used the spectrogram view in the sound analysis software RavenPro (version 1.5.0 Build 43 for Windows, 2017). The DFT size was 512 samples with an overlap of 50%, giving a resolution of ± 256 Hz. The spectrogram of each recording was viewed in a standard gamma II color scheme with a power threshold floor setting of 56 dB, although it should be noted that these recordings are not calibrated and this dB value is relative to the arbitrary reference value of the Raven software. For recordings with high background noise, the floor threshold was gradually raised to diminish noise and highlight avian acoustic communication. All reviews of the spectrograms and sound were conducted by one of us (KDK) who was experienced with the vocalizations of this bird community. Noise‐cancelling over‐ear headphones were used during review. Bird vocalizations, consisting of songs and calls, were identified to species. We tallied only vocalizations with a recognizable spectrogram that was clearly distinguishable by eye and ear from background noise. Two 10‐minute samples in 2018 occurred during substantial rain and were excluded from further analysis (see Approach 2 for additional details on quantifying and addressing sound from rain and wind). There were occasional high amplitude vocalizations that exceeded the sensitivity scale of the maximum amplitude that the recording system could record accurately (a phenomenon known as “clipping”), but clipped vocalizations could still be identified to species. The sound recordings were reviewed in random order to limit effects from listener bias and listener learning. The complete species annotations for all 410 10‐min recordings are depos in (Symes et al., 2021).

Each 10‐min recording was analyzed by counting the number of vocalizations (calls and songs) of each species present in the recording. Our objective was to recognize species and quantify their vocalization activity, so we did not attempt to distinguish between songs and calls, but we tested for the uniformity of vocalizations within species.

The duration and structure of bird vocalizations varied among species. For example, Red‐eyed Vireos (*Vireo olivaveus*) had a short but repeated song that included two elements over only about 700 ms. Black‐throated Blue Warblers had a song with several elements over about 1.5 s, whereas Winter Wrens (*Troglodytes hiemalis*) had songs of 5–10 s that consisted of a dozen or more elements per second. Operationally, we defined a vocalization event as an acoustic element separated from others by a pause of more than one second. This was partly subjective, so we characterized our operational definitions with sample sound recordings, associated spectrograms, and statistical analyses of mean duration and dominant frequency. Occasional incomplete vocalizations were still scored as one vocalization if they could be identified to species. Vocalizations of two mammals, red squirrels (*Tamiasciurus hudsonicus*) and Eastern chipmunks (*Tamias striatus*), were also recorded in the annotations of sound recordings from 2018.

#### 2.3.1 Repeatability of annotation

At the end of the annotation process, the same observer (KDK) blindly re‐annotated thirteen randomly selected 10‐min recordings. This allowed us to assess the consistency of the species lists and call counts.

### 2.4 Data distributions, transformations, and analyses

Species‐specific vocalization rates (number per 10‐min recording) had frequency distributions that were skewed toward higher vocalization rates (approximated gamma distributions). These distributions were well‐normalized with a square root transformation, which facilitated statistical analyses. The analyses are summarized in Table S2.

### 2.5 Research questions and statistical analyses

#### 2.5.1 Species lists and descriptions of vocal variation

Determining the list of species present in a site is among the most basic uses of annotated data and underlies many management and conservation decisions. We used the annotated data to generate an overall species list for Hubbard Brook that we could compare to decades of field observations from this well‐studied bird community (Holmes et al., 2021).

We quantified species‐specific patterns of vocalizations for the songs of the eight most common songbird species and compared them to determine how species were differentiated by duration and frequency. To select vocalizations for analysis, we isolated and analyzed a stratified random sample of recordings that consisted of two vocalizations per species from each of six recorders on each of two dates during 2016. Presumably, each recorder represented different individual birds. The two dates for each species × recorder combination were chosen as those that contained the most vocalizations for that species. We then selected two random numbers from 1 to *n*, where *n* was the number of vocalizations by that species in that recording. By manual review, we located those two vocalizations within recordings, noted the start and stop times to ± 0.01 s, and extracted the short segments of sound (snips) corresponding to the selected vocalization. This produced stratified random samples of 9–22 vocalizations per species (total of 157 snips) that we used to compare variation within vs. among species in the duration and character of vocalizations. We examined all spectrograms by eye to assess variation in vocalization characteristics within and among bird species and deposited the snip sound files at Symes et al. (2021). We could not readily calculate dominant frequency from these samples because most randomly selected bird vocalizations overlapped with vocalizations from other birds. Therefore, from the same sound recordings, we also attempted to locate two clean vocalizations per species (i.e., vocalizations that did not overlap with vocalizations of other birds, or other transient acoustic events such as from thunder, rain, or wind). It was not possible in each recording to find two clean vocalizations, but we obtained 2–22 clean vocalizations from seven species. We used *seewave* (Sueur et al., 2019) and *tuneR* (Ligges et al., 2018) functions in R Version 3.5.2 (R Core Team, 2017) to calculate the dominant frequency for each clean vocalization.

#### 2.5.2 Relationships among environmental variables, vocalization activity, and acoustic detection

Environmental variables such as rain, wind, cloud cover, barometric pressure, and temperature can impact avian physiology and behavior, as well as signal transmission and the probability of detecting a vocalization on a recording unit (Bruni et al., 2014). Understanding the interaction between vocalization and abiotic factors inform natural history (Bruni et al., 2014; Lengagne & Slater, 2002) and can have value for identifying the habitats and sampling windows that will be most valuable for observer‐based fieldwork.

We analyzed weather data that were collected in Hubbard Brook 0.2 to 2.4 km from our Hubbard Brook recorders and 16 km from our Jeffers Brook recorders. Weather data came from the publicly available Soil Climate and Analysis Network (SCAN site 2069, elevation 451 m asl) operated by the Natural Resources Conservation Service (NRCS) of the U.S. Department of Agriculture. We extracted data from each recording day for air temperature, precipitation, wind direction, wind speed, solar radiation, relative humidity, and dewpoint. All measurements were for 06:00 to 07:00 (which included our recording period of 06:20 to 06:30 local time, UTC‐4) except for relative humidity, which was an instantaneous measurement at 07:00. Measurements of average daily barometric pressure (and change in pressure from previous day) came from the Stagecoach Hill weather station in Plymouth, NH (43.74°N, 71.69°W, accessed via https://www.wunderground.com/dashboard/pws/KNHPLYMO5 on 29 May 2020), which was 21 and 35 km, respectively, from the acoustic recorders at Hubbard Brook and Jeffers Brook.

##### Quantification of rain and wind intensity

Rain and wind pose particular challenges for bioacoustics because they can affect both the rate of signaling in animals and the detectability of signals. We employed multiple approaches to quantifying background sound pressure from rain, wind, and water drops. The first approach was to reference hourly data from nearby weather stations (see above). A second approach was human review and evaluation of acoustic signatures associated with rain and wind (Towsey et al., 2012). Sound from water drops was ranked on a five‐tier scale by listening to the recordings and visualizing spectrograms: absence (0), drizzle to light (1), moderate and constant (2), hard rain (3), and very hard rain (4) (Figure S1). Wind, which tended to be audible but with a broadband spectral contribution below the visualization threshold, was ranked on a three‐tier auditory scale: absence (0), soft (1), or hard (2).

We also employed two statistical assessments of ambient sound pressure as recorded in the wave files: *A*_manual_ and *A*_automated_. Amplitude‐calibrated equipment is currently rare in terrestrial PAM. Our equipment was not amplitude calibrated and consequently, the measurements are proportional to sound pressure levels but cannot be represented as absolute values. Our calculations assumed that microphone sensitivity was approximately equal across recorders and across the duration of the recording season (verified by recording the same tone series using all recorders at the beginning and end of the seasons). For the calculation of *A*_manual_, we randomly selected 42 audio recordings from 2016 and manually identified (using the spectrogram view in RavenPro) a sample of one‐second sound snips without bird vocalizations from each recordings. To do so, we selected a random second within each minute and manually moved forward in the recording (by auditory review and examination of spectrograms) to locate the next one‐second interval that did not contain bird vocalizations, allowing us to sample the background throughout the recording. Sometimes, there were no one‐second intervals within the next minute without bird vocalizations. In those cases, we moved forward to the following minute. From 42 10‐minute sound files, we obtained 293 one‐second snips (available as WAV files at Symes et al., 2021): that is, 2–10 samples per recording of one second without bird vocalizations (mean = 7 per recording). For each snip, we used MATLAB to access the vector of raw acoustic sample values and calculated the root mean square (RMS) of sound pressure within each second (one snip) as the standard deviation of the raw sample values (*n* = 44,100 records in one second). The resulting data approximated a log‐normal distribution, so we used log_10_(RMS) for subsequent calculations. Our second statistical approach was an automated estimator of ambient sound pressure (*A*_automated_) that we were able to apply to all 410 10‐min recordings. The algorithm (implemented in MATLAB) calculated probability density functions for total sound pressure per second [log_10_(RMS)] in each minute of the recording (with a sliding frame that advanced by 0.1 s per step – yielding 580 1‐s sound snips per minute). We took the 10th percentile of the vectors of 580 snips as an operational metric of relative quiet in that minute and then calculated an average for the recording of these ten estimates of a quiet second (one estimate from each minute within the recording). MATLAB code for these analyses is available at https://github.com/MattAyres125/Estimator‐for‐ambient‐sound‐pressure.

We analyzed *A*_automated_ by ANOVA (JMP Pro 15.0, SAS Institute 2019) to estimate the relative contributions (percent total random variance) from dates, recorders, and minutes to the amplitude of background sound. The data frame included one value of *A*_automated_ for each minute of 410 10‐minute recordings (*n* = 4100 measurements). The ANOVA model included year (2016 and 2018), locations within year, and occasions within year. Locations and occasions were treated as random effects.

We calculated correlations across dates among all pairs of environmental factors (temperature, wind, ambient sound, etc.) and between environmental factors and bird vocalization rates.

#### 2.5.3 Community patterns in vocalization activity

Passive acoustic monitoring is well‐suited for revealing how species are associated with different habitats. Often, habitat affinity is described at a coarse scale (e.g., old growth forest, marshlands), with conservation decisions following comparably broad classes. But there can be substantial heterogeneity within recognized habitats due to, for example, diverse plant communities, topography, and proximity to water. Understanding where species spend time within preferred habitat types can help to identify and protect the most valuable areas within critical habitats.

We evaluated patterns in vocalization rates across two habitat types using our site comparison dataset, collected in 2016. We employed an ANOVA that included forest type (mature and mid‐aged), watershed (Jeffers Brook and Hubbard Brook), forest type × watershed, and date as fixed effects. To avoid concerns regarding spatial independence of recorders (Hurlbert, 1984), the data frame for the ANOVA was the average on each sample day of the 2–3 recorders within each forest type × watershed. Vocalizations per 10 min were square‐root transformed prior to analysis, which satisfied assumptions of homoscedasticity. Visual examination revealed no temporal autocorrelation to residuals.

Repeated sampling across 19 dates in 2018 permitted the construction of species accumulation curves to evaluate the completeness of species detections at each sampling location (see Approach 7).

#### 2.5.4 Timing of vocal activity

In mid‐ to high‐latitude systems, the annual timing of breeding events by birds can vary from year to year. For example, at Hubbard Brook, the annual variation in the initiation of first clutches by Black‐throated Blue Warblers varied by 20 days across 25 years (Lany et al., 2016). The annual timing of vocal activity would also be expected to vary among years, but data are limited (Buxton et al., 2016; Furnas & McGrann, 2018). The phenology of vocalization activity could be informative with respect to behavior, reproduction, and climatic patterns, and other environmental conditions. For example, the number of days of singing per season could be relatively constant from year‐to‐year or might vary depending on environmental conditions that influence the number and timing of clutches.

To evaluate phenological patterns, we plotted vocalization rates for each species by date across the breeding season.

#### 2.5.5 Correlations within species in vocalization activity

Within a season, there can be shifts in vocalization patterns due to breeding cycle, but there can also be daily variation around the trend. Daily vocalization activity can depend upon the weather (Bruni et al., 2014; Gottlander, 1987), the social environment (Fitzsimmons et al., 2008; Foote et al., 2011), the presence of predators, or other factors (Fitzsimmons et al., 2008; Foote et al., 2011; Stehelin & Lein, 2014; Valcu & Kempenaers, 2010; Xia et al., 2014). Weather tends to co‐vary over relatively large spatial scales, whereas social environments and predators tend to be more local. Therefore, we propose and test the hypothesis that weather will generate spatial correlations in day‐to‐day vocalization activity at the scale of tens of kilometers, whereas social interactions and predators predict correlations at the scale of hundreds of meters. We tested these predictions with analyses of spatial correlations in day‐to‐day vocalization rates. We used different analytical approaches for 2016 and 2018 because the spacing of the recorders and research questions were different.

With the data from 2016, we tested for species‐specific correlations in vocalization activity between the two stand ages within each forest type (recorders separated by ~150 m) and between the two watersheds (separated by ~16 km). For each of the 20 measurement dates, we calculated the mean of square root transformed vocalization rates for the 2–3 recorders in each age stand in each watershed (which largely normalized the distributions) and calculated the Pearson correlation coefficient of calling rates across days for the two sets of recorders in each watershed (separated by ~150 m). We then averaged the two stands within each watershed for each measurement date and calculated the Pearson correlation coefficient for vocalization rates across days between the two watersheds (separated by ~16 km). We estimated standard errors for the correlation coefficients as:

$$\text{SE}={\left(\frac{1‐r^{2}}{n‐2}\right)}^{0.5}$$

where *r* = correlation coefficient and *n* = sample size (Neter et al., 1985).

With the data from 2018, where we had a range of inter‐recorder distances, we were able to calculate continuous spline correlograms with 95% confidence intervals using the R package *ncf* (Bjornstad & Bjornstad, 2020). For these correlograms, we added data points representing the Pearson correlation coefficients vs. distance for all pairs of recorders; these data points were not independent because 10 recorders yielded 45 pairs. While these points did not influence the correlograms or the confidence intervals produced by *ncf*, they were plotted to facilitate data visualization. We omitted two dates with heavy rain and very low vocalizations (Figure 4) leaving 19 dates.

#### 2.5.6 Correlations among species in vocalization activity

The majority of species were detected throughout the window of dates that we sampled, indicating that we were sampling within the season of active calling, and not capturing arrivals or departures. Besides patterns within species, there could be correlations among species in day‐to‐day vocalization activity. Vocalizations would be positively correlated among species if they have similar responses to abiotic factors or predators (Nolen & Lucas, 2009). Alternatively, vocalizations could be negatively correlated if species have opposite reactions to abiotic factors such as heat or rain (e.g., rain may have more impact on bird species that use vocalizations that are short or structurally complex; Bruni et al., 2014).

We used a randomization test to evaluate interspecific correlations in day‐to‐day vocalization activity in the 2018 spatial dataset. We first calculated the mean of root‐transformed vocalizations per species on a given date and calculated the correlation matrix (Pearson's R) among all species pairs across 19 dates (excluding the two dates with heavy rain). To generate the null distribution, we again calculated correlations between all species pairs, first randomizing the call counts of both species with respect to date. This randomization was repeated 1000 times for each species pair to generate a distribution of correlation coefficients. We then compared the correlation coefficients of the actual data to the distribution of coefficients from the date‐randomized data to search for pairs of species that were more or less correlated than would be expected by chance.

To explore for natural groupings among species in vocalization behavior, we also used a principal components analysis to evaluate the correlation matrix of interspecies vocalization rates (square root‐transformed rates; rows as dates and columns as species). We then tested for correlations between the resulting principal components and environmental variables.

#### 2.5.7 Species accumulation and optimization of bioacoustic sampling schemes

We analyzed our 2018 data using EstimateS 9.1.0 Biodiversity Estimation Software (Colwell, 2013) to (1) estimate species accumulation curves for each individual recorder and for the aggregate of 10 recorders, and (2) estimate total species richness represented in the aggregate of 10 recorders. Species accumulation curves described the expected number of species in *t* pooled samples (equation 17 in Colwell, 2013). In addition to being a description of the vocalizing community, the relationship between number of species detected and sampling effort is fundamental to optimizing the design of bioacoustic studies.

We then evaluated how estimates of species richness would be affected by alternative possible sampling schemes that vary the number of locations and dates analyzed. Our 2018 data included 10 locations × 19 occasions, or 190 annotated 10‐min sound recordings. With the same analysis effort, the theoretically possible sampling strategies would include one location with 190 occasions, two locations with 95 occasions, etc. To estimate the expected species richness with all possible strategies, we created a simulated data set from our data (Appendix S2) that included 190 sampling occasions for each of 10 sampling locations. From this extended data set, we drew replicated (*n* = 1000) random samples for each possible combination of locations and occasions (from one location on one occasion) to 10 locations on 190 occasions. We calculated the average species richness (from 1000 replicated random draws) for each combination of number of locations and occasions.

## 3 RESULTS

### 3.1 Species lists and descriptions of vocal variation

Our sample of 410 10‐min sound recordings included 129,866 vocalizations from 44 bird species and two mammal species (Table 1). The total number of vocalizations per 10‐min recording ranged from 3 to 1567 with a median of 303 and standard deviation of 208. The total number of species detected per 10‐min recording ranged from 1 to 12 species with a median of 5. In 2016, Red‐eyed Vireos accounted for 64.2% of the vocalizations, and the 10 most prevalent species accounted for 98.2% of the vocalizations (Table 1). In the 2018 dataset, Red‐eyed Vireos accounted for 50.2% of the vocalizations, with the 10 most prevalent species accounting for 90.4% of the vocalizations (Table 1). The full set of annotations are provided in (Symes et al., 2021) as the number of vocalizations by each of 46 species in each of 410 10‐min recordings.

**TABLE 1:** Total number of songbird vocalizations annotated from sound recordings collected in 2016 (200 10‐min recordings at Hubbard Brook and Jeffers Brook, combined) and 2018 (210 10‐min recordings at Hubbard Brook). Species are sorted by total vocalizations recorded

Species common name	Code	Total vocalizations	
		2016	2018
Red‐eyed Vireo	REVI	46,817	26,998
Black‐throated Blue Warbler	BTBW	6890	4316
Black‐throated Green Warbler	BTNW	5399	4096
Ovenbird	OVEN	3781	4192
Hermit Thrush	HETH	4051	1824
Red‐breasted Nuthatch	RBNU	32	2804
American Redstart	AMRE	2278	233
Blue‐headed Vireo	BHVI	427	1995
Swainson's Thrush	SWTH	880	1313
Red Squirrel^a^	RESQ		910
Yellow‐rumped Warbler	YRWA	48	847
Winter Wren	WIWR	514	215
Golden‐crowned Kinglet	GCKI	511	172
Black‐capped Chickadee	BCCH	92	561
Yellow‐bellied Sapsucker	YBSA	299	304
Eastern Wood‐Pewee	EWPE	0	542
Black‐and‐white Warbler	BAWW	73	459
Scarlet Tanager	SCTA	48	338
Blue Jay	BLJA	62	296
Magnolia Warbler	MAWA	0	328
White‐breasted Nuthatch	WBNU	0	315
Blackburnian Warbler	BLWA	7	307
Dark‐eyed Junco	DEJU	0	249
Hairy Woodpecker	HAWO	68	177
American Robin	AMRO	218	18
Pine Siskin	PISI	0	220
Brown Creeper	BRCR	87	106
Cape May Warbler	CMWA	34	159
Rose‐breasted Grosbeak	RBGR	56	100
Red Crossbill	RECR	0	92
Louisiana Waterthrush	LOWA	64	0
Purple Finch	PUFI	24	39
Downy Woodpecker	DOWO	48	1
Veery	VEER	0	31
Wood Thrush	WOTH	0	26
Eastern Chipmunk^a^	EACH		24
Ruby‐throated Hummingbird	RTHU	21	1
Pileated Woodpecker	PIWO	10	11
Alder Flycatcher	ALFL	0	18
American Goldfinch	AMGO	0	14
Cedar Waxwing	CEDW	0	13
Blackpoll Warbler	BLPW	0	8
Canada Warbler	CAWA	0	5
Pine Warbler	PIWA	0	4
Common Loon	COLO	3	0
Great‐crested Flycatcher	GCFL	2	0
Northern Parula	NOPA	0	2

^a^ Only enumerated in 2018.

Vocalizations were relatively stereotypical within species (Figure S2), including in duration, frequency, and pattern (Table 2). The longest vocalizations were produced by Winter Wrens (mean = 6.2 s). The remaining species had vocalizations ranging from 0.6 s (Red‐eyed Vireo) to 2.7 s (Ovenbird; *Seiurus aurocapilla*). The peak frequency of vocalizations ranged from 2.9 to 5.5 kHz (Swainson's Thrush *(Catharus ustulatus)* and American Redstart (*Setophaga ruticilla*), respectively). The relatively long song of the Winter Wren meant that their vocalizations usually overlapped with other species and so we were unable to quantify peak frequency for this species. While the highest and lowest frequency species in the community had little frequency overlap, all of the common species used frequencies that overlapped with at least some of the other common species.

**TABLE 2:** Attributes of vocalizations of eight common species of breeding songbirds at Hubbard Brook. See Table 1 for full species names

Species	Code	*n* ^a^	Duration (s)		Peak frequency (kHz)	
			Mean	SD	Mean	SD
American Redstart	AMRE	22, 7	1.16	0.30	5.46	1.17
Black‐throated Blue Warbler	BTBW	22, 13	1.53	0.29	4.45	0.13
Black‐throated Green Warbler	BTNW	18, 10	1.46	0.22	4.93	0.57
Hermit Thrush	HETH	20, 11	1.39	0.38	3.63	0.68
Ovenbird	OVEN	20, 8	2.67	0.53	4.24	0.22
Red‐eyed Vireo	REVI	24, 22	0.61	0.13	3.37	0.41
Swainson's Thrush	SWTH	9, 2	1.19	0.28	2.93	0.49
Winter Wren	WIWR	22, 0	6.15	1.25		

^a^ Number of randomly selected vocalizations used to estimate (duration, peak frequency).

There were no cases where a species was added or lost in a blind second annotation and there was high repeatability in the counts of vocalizations per species per recordings (*r* ^2^ = 0.92 to 0.99; *n* = 13, depending on the species; Figure S3). The modest differences between replicate counts from the same sound recordings resulted from low amplitude vocalizations that were on the edge of detectability and were counted in one sample but not the other.

### 3.2 How vocalization rates are impacted by abiotic factors

**FIGURE 2:**
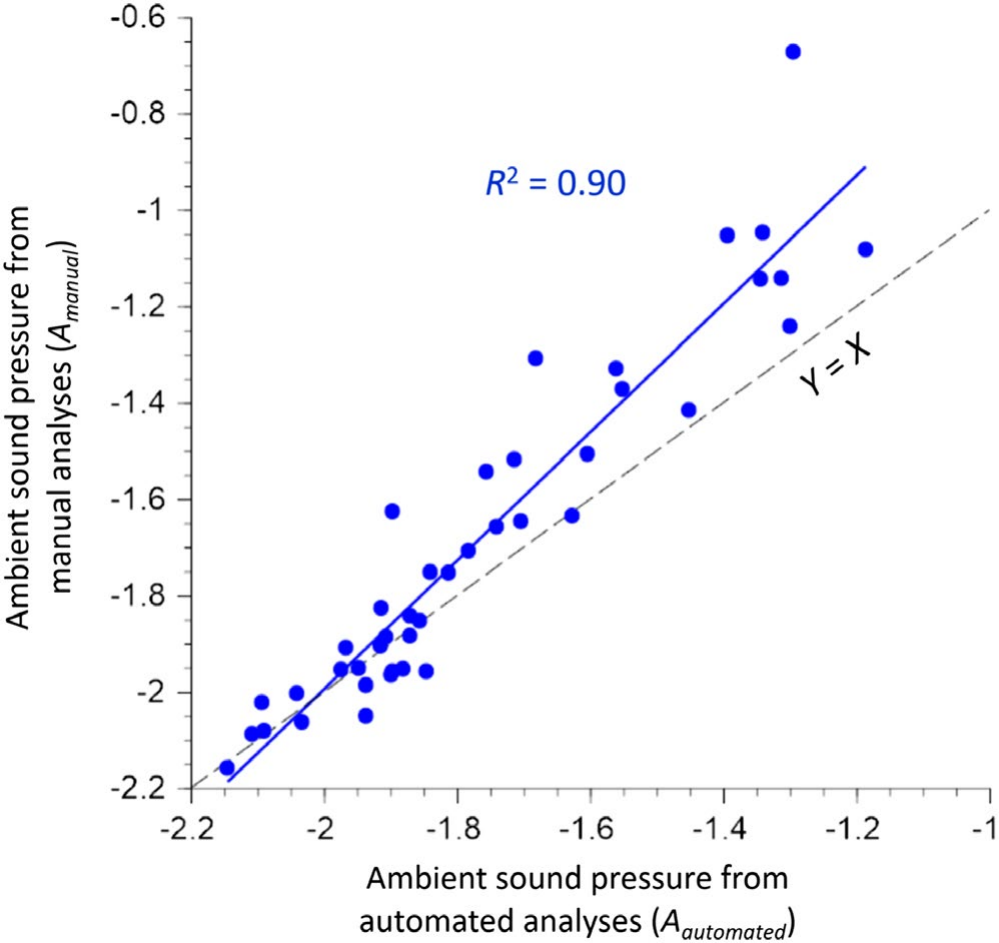
Comparison of estimates of ambient sound pressure from (1) manually identified seconds with no bird vocalizations (*y*‐axis) to (2) automated analysis of ambient sound pressure (*x*‐axis). Dashes represent the line of equality. Units are log_10_(RMS)

Air temperatures during our acoustic sampling ranged from 7 to 20°C (mean ± SD = 15 ± 4°C; Table S3). Two of 41 sampling days had substantial rainfall during the hour of the recording (2.6 and 3.5 mm/h on 04 June and 28 June 2018), and one day had trace precipitation (23 May 2018). Conditions were generally calm at the time of our recordings (windspeed <1 km/h on 63% of days), but 10% of the days had average wind speeds >2 km/h (Table S3). Solar radiation (chiefly sun vs. clouds) and barometric pressure were variable among sampling occasions. Although there were only three of 41 sampling occasions during which precipitation was recorded at the nearby weather station, recordings from many days included dripping sounds (Table 2 and Figure S1), apparently the result of condensation in the canopy. Such dripping was more pronounced when temperatures approached the dewpoint and when there was wind. The sound pressure in randomly chosen seconds when no birds were vocalizing varied among sampling occasions and was well correlated with automated quantification of ambient sound pressure (Figure 2). The divergence between the metrics at high sound pressure levels likely reflects the fact when focal seconds were chosen, we selected only quiet seconds (up to 10, but often fewer). The automated analysis identified the quietest 10th percentile, which may still contain some acoustic events when recordings had substantial acoustic activity. The automated quantification could be performed for all 410 recordings. Ambient sound pressure was highly variable among days, with much less variation among sampling locations or among minutes within sampling occasions; 85% of the random variance in background sound pressure was among days versus only 4% and 11% among locations on a day and among minutes within 10‐minute recordings, respectively. The two rain days in 2018 had 17‐ to 29‐fold more background sound pressure than the quietest day; the other 19 days differed by no more than 4‐fold in background sound pressure (Figure S4).

The number of bird vocalizations from all species that was detected per recorder per 10‐min sampling occasion was negatively correlated in both years with a set of intercorrelated variables related to ambient sound: wind speed, wind sounds, and dripping sounds (Tables 3 and 4). Overall vocalization activity per day was negatively related in both years to wind speed and ambient sound (Table 5). There was a weaker positive association with barometric pressure (significant in 2016 but not 2018). For the most common species, vocalization activity and date were often correlated, but this direction was not consistent between years for all common species except Ovenbirds (Table 5). There were some additional correlations between vocalization activity and environmental variables, but other than relations with wind speed and ambient sound they were infrequent and inconsistent (Table 5).

**TABLE 3:** Correlations among environmental variables and total bird vocalizations at Hubbard Brook in 2016. *N* = 20 dates, 10 recording sites. Correlations with |*r*| > .45, .56, and .68 (in bold) correspond to approximate *p*‐values of .05, .01, and .001, respectively

	JulianDate	Temperature	Wind speed	Solar radiation	Relative humidity	Temp‐dewpoint	Barometric pressure	delta pressure	Dripping sound	Wind sound	Ambient sound
JulianDate	1.00										
Temperature	**0.61**	1.00									
Wind speed	0.00	−0.34	1.00								
Solar radiation	**−0.76**	**−0.57**	−0.06	1.00							
Relative humidity	0.44	0.26	−0.26	−0.37	1.00						
Temp‐dewpoint	**−0.47**	−0.24	0.27	0.38	**−0.96**	1.00					
Barometric pressure	0.13	0.19	**−0.60**	0.06	−0.15	0.07	1.00				
Delta pressure	0.05	−0.10	0.20	−0.10	−0.36	0.38	−0.01	1.00			
Dripping sound	−0.04	−0.35	**0.81**	−0.09	−0.01	−0.01	**−0.61**	−0.09	1.00		
Wind sound	−0.30	−0.34	**0.46**	0.18	**−0.55**	**0.56**	−0.19	−0.08	**0.55**	1.00	
Ambient sound energy	−0.12	−0.34	**0.77**	0.07	−0.27	0.27	**−0.49**	0.23	**0.80**	**0.58**	1.00
Bird vocalizations	−0.45	0.09	**−0.64**	0.38	−0.13	0.19	**0.46**	0.00	**−0.78**	−0.33	**−0.61**

**TABLE 4:** Correlations among environmental variables and three measures of bird vocalizations at Hubbard Brook in 2018. *N* = 19 dates, 10 recording sites. Correlations with |*r*| > .45, .57, and .69 (in bold) correspond to approximate *p*‐values of .05, .01, and .001, respectively. Two dates (4 and 28 June) with rain during the the recording were excluded from the analysis

	JulianDate	Temperature	Wind speed	Solar radiation	Relative humidity	Temp‐dewpoint	Barometric pressure	delta pressure	Dripping sound	Wind sound	Ambient sound	Bird vocalizations (average)
JulianDate	1.00											
Temperature	0.41	1.00										
Wind speed	0.11	0.09	1.00									
Solar radiation	−0.12	−0.14	0.16	1.00								
Relative humidity	−0.05	0.25	−0.03	−0.43	1.00							
Temp‐dewpoint	0.05	−0.07	−0.13	0.33	**−0.91**	1.00						
Barometric pressure	0.33	−0.18	−0.17	0.01	−0.18	0.11	1.00					
delta pressure	0.42	−0.20	0.12	0.11	−0.14	0.06	**0.66**	1.00				
Dripping sound	−0.05	−0.05	**0.68**	−0.30	**0.48**	**−0.59**	−0.19	0.03	1.00			
Wind sound	0.01	−0.07	**0.68**	−0.09	−0.07	−0.05	0.09	−0.01	**0.72**	1.00		
Ambient sound energy	−0.10	0.06	**0.69**	−0.24	**0.53**	**−0.67**	−0.19	−0.03	**0.92**	**0.65**	1.00	
Bird vocalizations (average)	0.10	0.28	**−0.61**	0.18	0.06	0.04	0.25	0.08	**−0.58**	**−0.56**	**−0.58**	
Bird vocalizations (PC‐1)	**−0.70**	−0.22	−0.25	0.11	−0.10	0.16	−0.08	−0.20	−0.28	−0.18	−0.23	1.00
Bird vocalizations (PC‐2)	0.34	0.27	**−0.61**	0.21	0.06	0.06	0.34	0.24	**−0.61**	**−0.63**	**−0.60**	0.00

**TABLE 5:** Correlations between vocalization rates of individual songbird species and environmental variables (*N* = 20 dates for 2016, *N* = 19 dates for 2018). Vocalization rates are the average for each date of square‐root transformed rates for all recorders at Hubbard Brook (5 in 2016 and 10 in 2018). Analyses for 2018 exclude two rainy dates. See Tables 3 and 4 for correlations among environmental variables

Species	Year	Pearson correlation coefficients							
		Julian date	Temper‐ature	Wind speed	Solar radiation	Relative humidity	Barometric pressure	Change in pressure	Ambient sound
All	2016	−0.45	0.09	**−0.64**	0.38	−0.13	**0.46**	0.00	**−0.61**
All	2018	0.10	0.28	**−0.61**	0.18	0.06	0.25	0.08	**−0.58**
REVI	2016	**−0.50**	−0.01	**−0.49**	**0.68**	−0.30	0.38	−0.20	−0.36
REVI	2018	0.39	**0.52**	−0.30	0.16	0.12	0.15	0.13	−0.35
BTBW	2016	0.37	**0.64**	**−0.66**	−0.19	0.29	**0.55**	−0.17	**−0.70**
BTBW	2018	−0.37	−0.05	−0.30	0.02	0.17	0.04	−0.09	−0.13
BTNW	2016	**0.59**	0.44	−0.21	−0.26	**0.52**	0.11	−0.04	−0.30
BTNW	2018	**−0.65**	−0.21	−0.31	0.34	−0.23	−0.03	−0.07	−0.42
OVEN	2016	**−0.57**	−0.07	−0.19	0.33	−0.37	0.16	−0.01	−0.37
OVEN	2018	**−0.51**	−0.04	**−0.49**	0.19	−0.06	0.17	0.12	−0.36
HETH	2016	0.13	0.01	−0.34	−0.14	0.05	−0.04	0.40	−0.29
HETH	2018	0.25	−0.03	−0.38	−0.05	0.15	0.05	0.20	−0.23
AMRE	2016	**−0.65**	−0.23	−0.31	0.39	−0.39	0.43	0.11	−0.28
RBNU	2018	**0.60**	0.18	−0.14	0.06	−0.18	0.32	0.01	−0.27
BHVI	2018	−0.19	−0.16	−0.31	−0.04	−0.28	0.18	−0.07	−0.46
SWTH	2018	**0.70**	0.32	−0.08	−0.03	−0.01	0.37	0.35	−0.16
YRWA	2018	−0.05	0.13	−0.38	0.11	−0.13	0.04	−0.08	−0.45

Note

Bold‐face indicates statistical significance (uncorrected for multiple comparisons). Critical values for *p* < .05, *p* < .01, and *p* < .001 ≈ correlation coefficients of .45, .57, and .69.

### 3.3 Community patterns in vocalization activity

**FIGURE 3:**
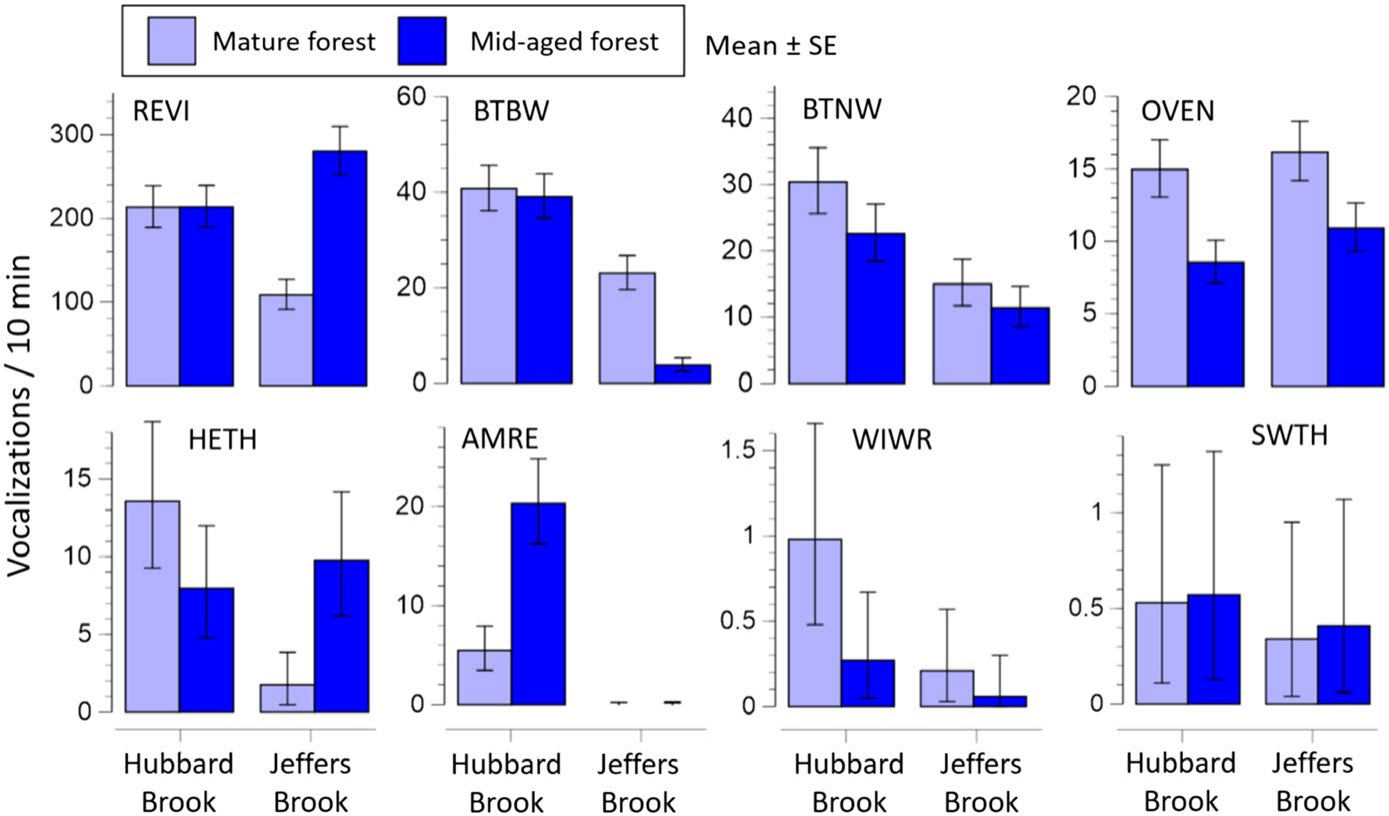
Vocalization rate of eight species of songbirds in the Hubbard Brook and Jeffers Brook watersheds, NH, in 2016. Means and SEs are from 20 dates × 2–3 recorders each in mature forest and mid‐aged forest in the two forested watersheds (Figure 1, Table 1). Corresponding ANOVAs are in Table S2. REVI = Red‐eyed Vireo, BTBW = Black‐throated Blue Warbler, BTNW = Black‐throated Green Warbler, OVEN = Ovenbird, HETH = Hermit Thrush, AMRE = American Redstart, WIWR = Winter Wren, and SWTH = Swainson's Thrush. Note different scales on vertical axes

Most bird species occurred in recordings from both Hubbard Brook and Jeffers Brook, but some species were detected primarily at one location (Figure 3, Table S4). The elevation of Jeffers Brook is approximately 100 m lower than Hubbard Brook, but the sites were otherwise similar in vegetation and land use history. Despite this apparent ecological similarity, American Redstarts and Black‐throated Blue Warblers were regularly detected at Hubbard Brook and almost never detected at Jeffers Brook, while American Robins (*Turdus migratorius*) were detected almost exclusively at Jeffers Brook.

There were clear associations between species and forest age, with Ovenbirds and Winter Wrens detected at higher rates in mature forests, while American Redstarts were more commonly detected in middle‐aged forest (Figure 3, Table S4).

### 3.4 Timing of vocalization activity

**FIGURE 4:**
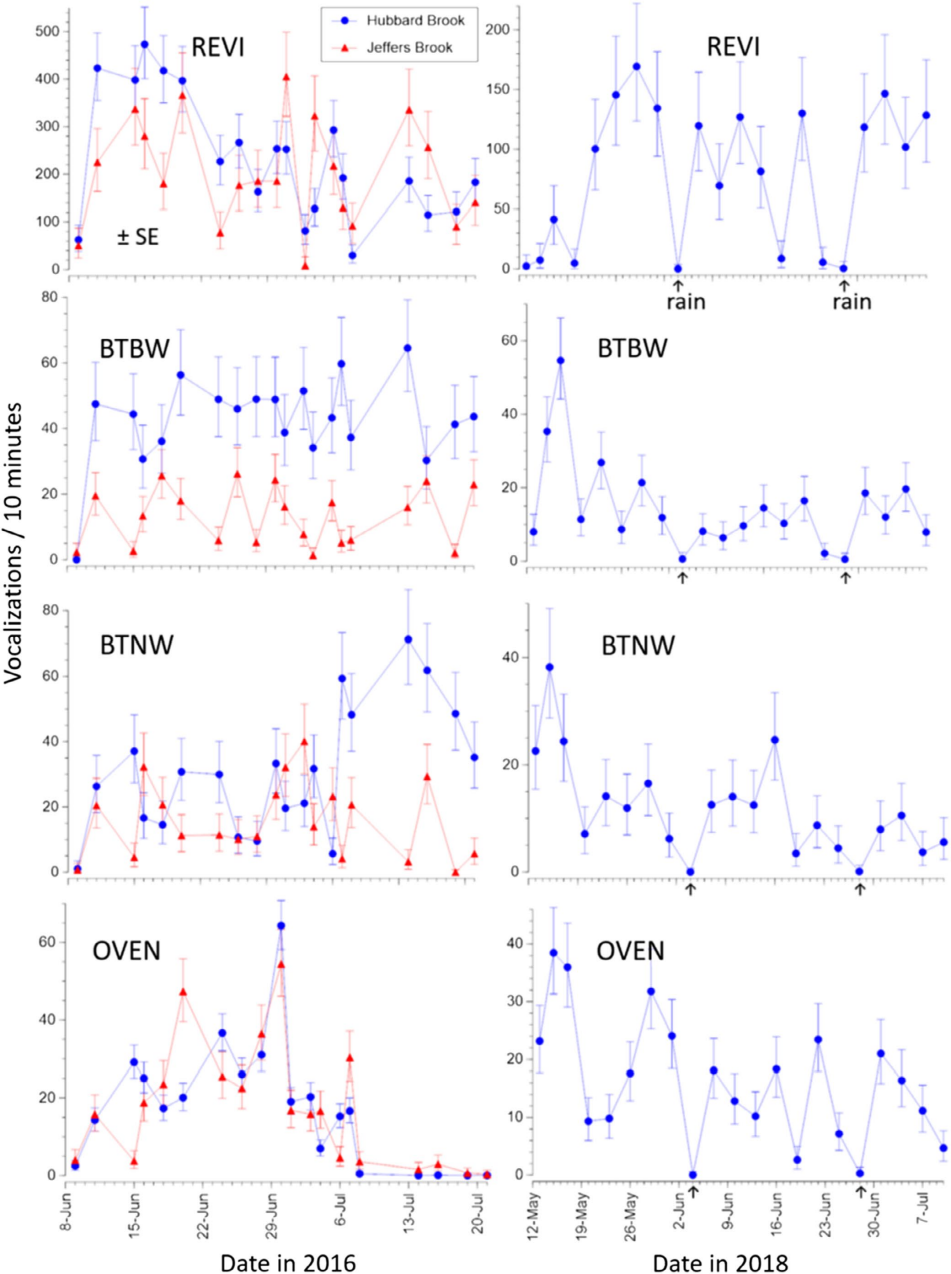
Vocalization rate of four nesting songbird species at Hubbard Brook and Jeffers Brook during two breeding seasons. Data are based on 10‐min recordings made about 1 h after sunrise (06:20 – 06:30 EDT) through each season. *N* = 5, 6, or 10 recorders (Hubbard Brook 2016, Jeffers Brook 2016, Hubbard Brook 2018, respectively). REVI = Red‐eyed Vireos, BTBW = Black‐throated Blue Warblers, BTNW = Black‐throated Green Warblers, and OVEN = Ovenbird. Two dates in 2018 with heavy rain during the recording time are indicated with arrows on the x‐axes. Note that measurements started later and ran later in 2016 than in 2018

Data from 2016 and 2018 were recorded in different nearby locations. In both years, most of the bird species were conspicuously vocal throughout our sampling window of 6–8 weeks, but species‐specific vocalization rates frequently varied by two‐fold or more among mornings separated by just a few days (Figure 4). In 2016, fluctuations in daily vocalization rates activity were quite concordant between Hubbard Brook and Jeffers Brook in 2016 (left‐hand column in Figure 4). In 2018, the earlier recordings captured comparatively high activity from Black‐throated Blue warblers, Black‐throated Green Warblers, and Ovenbirds, and comparatively low activity from Red‐eyed Vireos.

### 3.5 Correlations within species in vocalization activity

**FIGURE 5:**
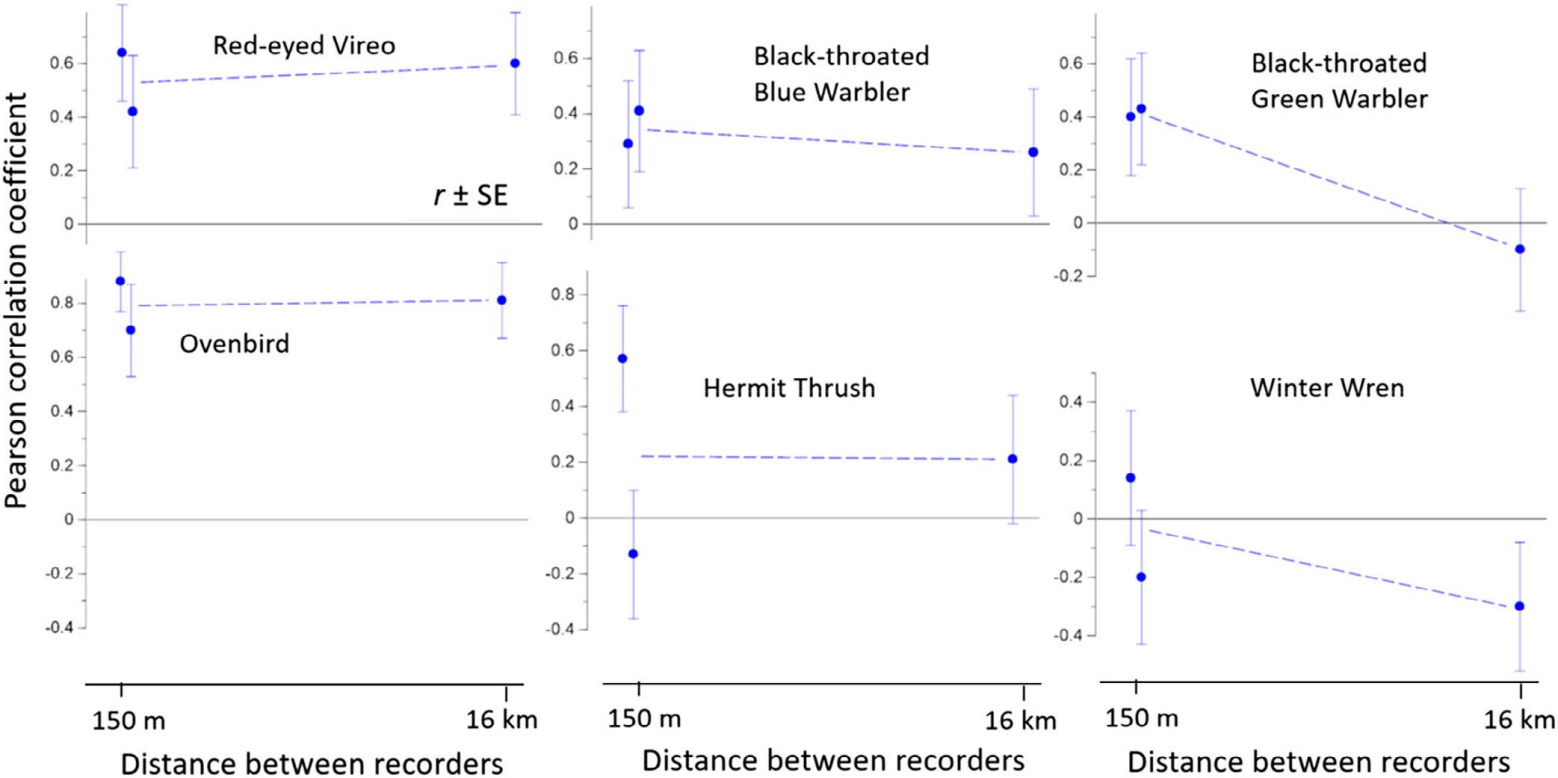
Intraspecific correlations in vocalization rates of six common bird species at two distances (150 m and 16 km; *n* = 20 dates in 2016). *Y*‐axis is the average Pearson correlation coefficient for the corresponding species and distance. The two points in each panel at 150 m show the average correlation coefficient from Hubbard Brook and Jeffers Brook. The one point at 16 km shows the correlation coefficient for Hubbard Brook versus Jeffers Brook

To further evaluate intraspecific spatial correlation in vocal activity, we tested for correlated vocalization dynamics both between habitats (forest age) within a site and between watersheds (Figure 5). For some species (Red‐eyed Vireo, Ovenbird, and perhaps Black‐throated Blue Warbler), daily vocal activity was correlated across recording sites, even when the sites were separated by more than 10 km. The correlations for Ovenbirds were particularly high (*r* ≈ .80). Recording sites separated by only about 150 m were generally no more similar than those separated by about 16 km. However, the daily vocalization rates of Black‐throated Green Warblers (Figure 5, upper right) were more correlated among nearby sites than distant sites.

**FIGURE 6:**
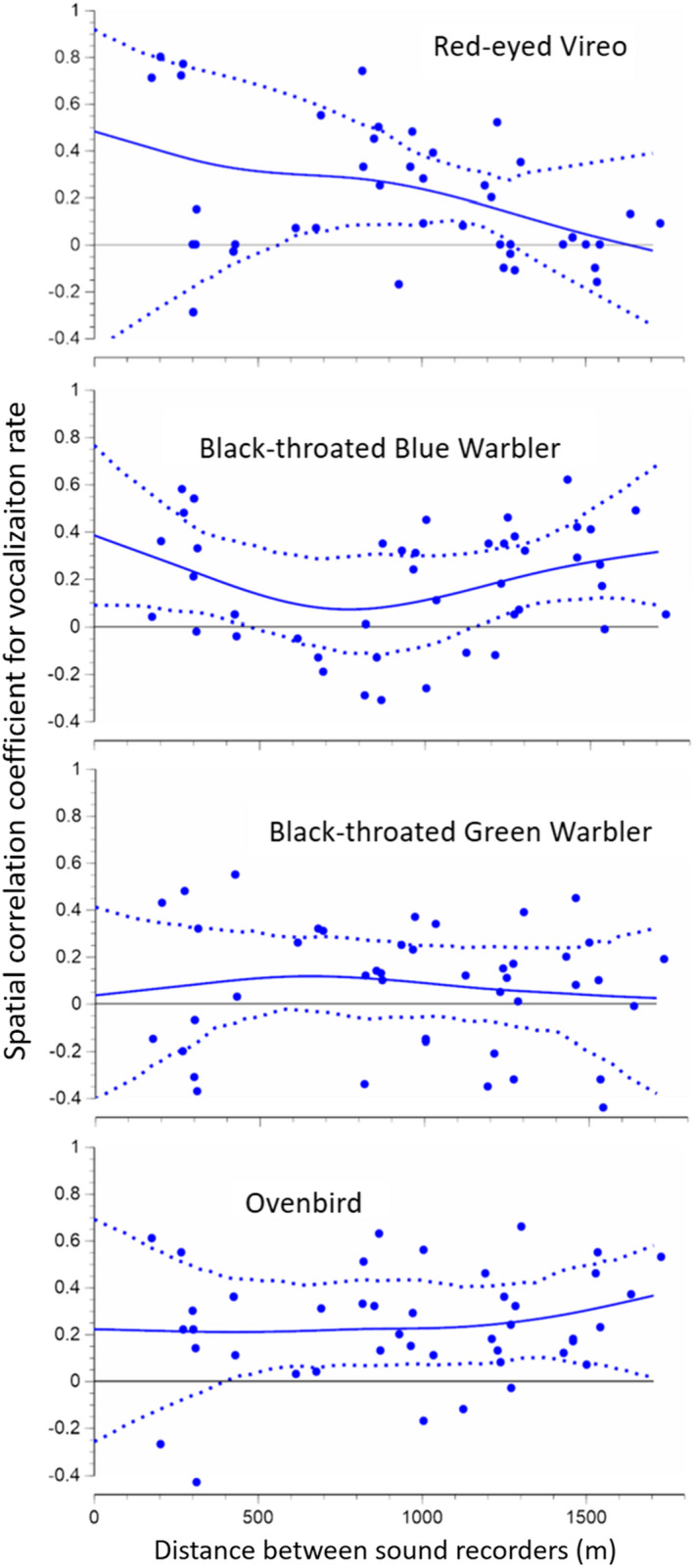
Intraspecific correlations in vocalization rates of four songbird species as a function of distance between recording locations (*n* = 19 dates × 10 locations in Hubbard Brook in 2018). Smooth lines indicate the nonparametric autocorrelation functions with 95% confidence intervals (Bjornstad & Falk, 2001). Points represent all pairs of recording locations

The data from 2018 permitted evaluation of spatial correlations in daily vocalization rate at the finer scale of 200–1500 m. Examination of georeferenced animations of daily vocalization rates across the study area (Appendix S1) suggested modest spatial correlation that depended on the species. Further resolution was permitted by spatial correlograms (Figure 6). Similar to the 2016 data, there was evidence of spatial correlations in vocalization rates, with Ovenbirds again showing particularly strongly correlated dynamics. However, with the possible exception of Red‐eyed Vireos, there was little evidence for elevated correlations among nearby locations.

### 3.6 Correlations among species in vocalization activity

**FIGURE 7:**
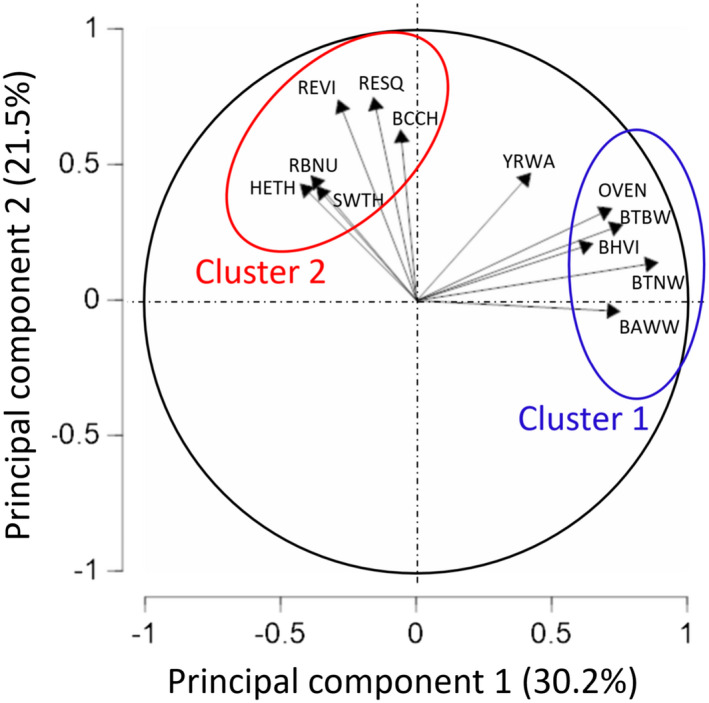
Principal components analysis of variation among days in the vocalization rate of 12 songbird species at Hubbard Brook. Species codes follow Table 2

Many species pairs had correlated peaks and troughs in daily vocalization rates (Table 6). The 66 pairwise correlations were disproportionately positive (45 correlations were positive, 10 were significant; 21 correlations were negative, none significant). The overall mean correlation was *r* = .14 with a SD = 0.29. There were two clusters of covarying species, and these tended to be negatively correlated with each other (note the positive correlations in the upper left and lower right of Table 6, and the negative correlations in the center left of the matrix). A principal components analysis of the call rate of 12 species across 19 dates explained 52% of the variation with two axes (Table S5). One cluster of species, loading positively on PC1 included Black‐throated Green Warbler, Black‐throated Blue Warbler, Ovenbird, Black‐and‐white Warbler (*Mniotilta varia*), and Blue‐headed Vireo (*Vireo solitarius)*. The second cluster of species, loading negatively on PC1, and more strongly on PC2, included Red‐eyed Vireo, red squirrel, Black‐capped Chickadee, Red‐breasted Nuthatch (*Sitta canadensis*), and Swainson's Thrush (Figure 7). When compared with environmental variables, the first principal component was positively correlated with date, while the second principal component was negatively correlated with ambient sound pressure (Table 4, bottom rows).

**TABLE 6:** Pairwise correlations in average vocalization activity between bird species at Hubbard Brook in 2018 (*N* = 19 days). Entries are Pearson correlation coefficients. Asterisks correspond to approximate significance levels from randomization tests [*p* < .05 (*) or *p* < .01 (**)]. Positive correlations are indicated with blue, negative with red. See Table 1 for complete species names

	BTBW	BTNW	OVEN	BAWW	BHVI	YRWA	REVI	HETH	SWTH	RBNU	BCCH
BTNW	0.53*										
OVEN	0.61**	0.72**									
BAWW	0.67**	0.57*	0.28								
BHVI	0.53*	0.56*	0.41	0.48							
YRWA	0.34	0.42	0.35	0.27	0.12						
REVI	0.04	−0.16	0.12	−0.22	−0.24	0.36					
HETH	−0.17	−0.36	−0.02	−0.22	−0.18	−0.23	0.47*				
SWTH	0.06	−0.35	−0.22	−0.13	−0.22	0.17	0.46*	0.04			
RBNU	−0.09	−0.34	−0.35	−0.15	0.27	−0.13	0.27	0.19	0.49		
BCCH	0.12	0.11	0.18	−0.12	0.14	−0.06	0.29	0.34	0.02	0.34	
RESQ	−0.14	0.06	0.11	−0.27	0.08	0.41	0.41	0.35	0.19	0.28	0.47

### 3.7 Species accumulation and optimization of bioacoustic sampling schemes

**FIGURE 8:**
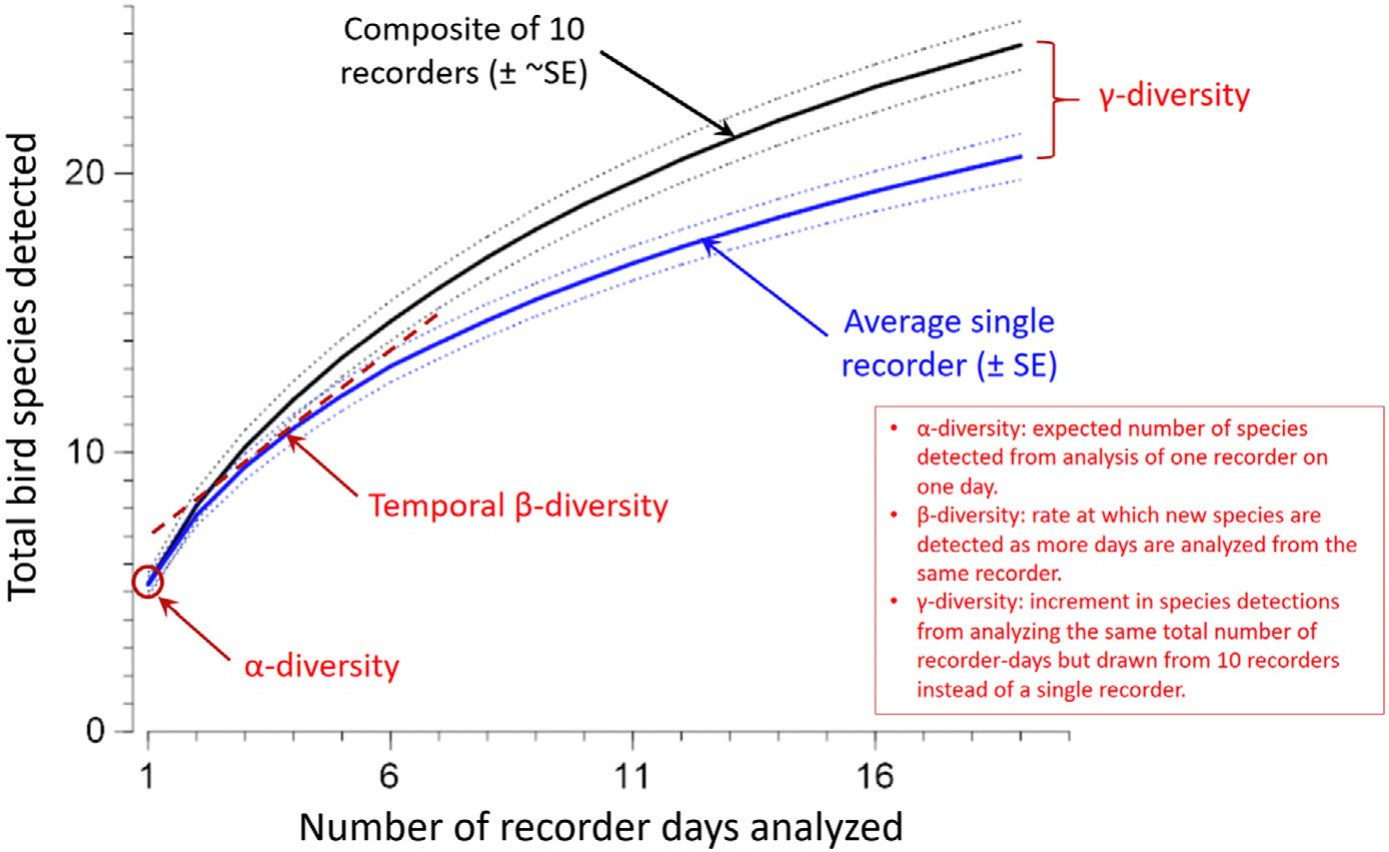
Relationship between total number of bird species detected via passive acoustic monitoring and the number of recorder days that were analyzed, as measured at Hubbard Brook. The blue curve indicates the average species accumulation for a single location. The black curve indicates the species accumulation for an equal number of samples randomly drawn from any of the 10 locations

The average number of species detected in one 10‐minute sample at one recording location was 5–6 species (α‐diversity, Figure 8). The number of new detections at an average location increased to about 13 species with 7 days of sampling (β‐ diversity, Figure 8). The expected total number of species detections increased from about 20 to 25 species if 19 ten‐min samples were drawn from 10 locations vs. all from the same location (γ‐diversity in Figure 8).

As anticipated, more species were detected when we sampled additional days and included additional recording sites within the habitat matrix (Figures S4 and S5). However, adding locations resulted in more species per unit of analysis effort than did adding more days (Figure 8). In our study system, the expected species detection curve saturates at 30 species when sampling in one location and at 41 species when the same total sampling time was distributed across ten locations (Figure 8).

## 4 DISCUSSION

Our examination of bird vocalization patterns from multiple sites, years, and recording units generated knowledge of the study system, as well as insights regarding methods. The species lists from acoustic data (Table 1) were largely congruent with decades of observer‐based field studies (see Holmes & Likens, 2016). For example, the five species responsible for 79–89% of all recorded vocalizations are the most abundant breeding birds in this location and the rest of our species list (Table 1) nearly completes the well‐refined list of breeding birds in Hubbard Brook Experimental Forest (Holmes & Sherry, 2001; Holmes et al., 1986). However, several species known to occur in the forest were absent from the acoustic data including Common Ravens (*Corvus corax*), several hawks (all species with large home ranges and low vocalization rates), Chimney Swifts (*Chaetura pelagica*, which forage above the canopy and have low amplitude calls), Ruffed Grouse (*Bonasa umbellus*) and Barred Owls (*Strix varia*; a species with primarily nocturnal calling that was not captured by dawn recordings). Longer duration recordings may enhance the representation of species that are active at other times of day. Unexpected detections in our recordings included an Alder Flycatcher (*Empidonax alnorum*; an apparent itinerant that was only detected at one location on one day) and a Common Loon (*Gavia immer*; likely a flyover). In cases with published call descriptions, there was strong alignment between the calls measured here and previously published measurements (Rivers & Kroodsma, 2000).

### 4.1 Environmental variables and ambient sound

We evaluated multiple environmental variables for their relations with vocalization activity. The literature includes numerous reports of such relationships. For example, sunlight (Miller, 2006; Thomas et al., 2002), moonlight (York et al., 2014), temperature (Garson & Hunter, 1979; Gottlander, 1987; Thomas, 1999), and atmospheric pressure (Prevost, 2016) can affect signaling activity. Wind and rain can interact to affect vocalization activity in a variety of bird species, ranging from King Penguins to Grasshopper Sparrows (Lengagne et al., 1999; Lenske & La, 2014; Prevost, 2016). In our studies, the only environmental variable with notable effects was background sound, which was primarily due to the sound of water dripping from the canopy.

Establishing statistical criteria for identifying recordings with high ambient sound (“noise” from the perspective of animal vocalizations) provides an objective way to filter recordings for analysis and identify how species respond to background noise. The automated approach to assessing ambient sound provided results that were highly correlated with the manual approach (Figure 2) and can readily be applied to large numbers of sound recordings. In our study system, some bird species showed greater declines in vocalization when ambient sound was relatively high (Table 4, Figure 7). High ambient sound can reduce the number of detected vocalizations by changing behavior or by changing the detectability of vocalizations. In our study, we detected fewer vocalization during rain, even though we excluded recordings with the most intense background sound. The fact that light rain days included fewer vocalizations that rain‐free days likely reflect the fact species are reducing their calling activity rather than calling and failing to be detected. However, changes in vocalization rate and changes in detectability could both contribute to the observed relationship between background sound and vocalization rate. Indeed, the detectability and vocalization rate may be correlated if species that are less detectible to receivers (and recorders) are also less likely less to attempt vocalization with high background sound. Further study would be needed to test whether species with low calling rates under high ambient sound share similar acoustic signals, such as lower frequency, lower amplitude, or narrower bandwidth (Snell‐Rood, 2012). Ambient sound could also influence the nature of vocalizations. For example Mountain Chickadees (*Poecile gambeli*) in Colorado tended to sing more and call less in noisier environments (LaZerte et al., 2017).

### 4.2 Variation in signaling activity across space and time

In the third approach, we compared vocal activity in two watersheds with similar forest composition and land use history. Despite the proximity and similarity of these sites, several bird species were common in one site and rare or absent in the other (Figure 3). These findings underscore the value of replicating locations within a habitat and not assuming that a single forest site is representative of similar nearby habitats.

Signaling is risky, time consuming, and metabolically costly (Falk et al., 2015; Godin & McDonough, 2003; Prestwich & Walker, 1981; Symes et al., 2015; Taigen & Wells, 1985). Although the characteristics of the individual signals have been well‐studied, much less is known about how singing activity varies from day‐to‐day or season‐to season. Additional sampling would be required to test for the stability in our study system of seasonal timing across years (Method 4). Most of the species that we studied displayed conspicuous peaks and troughs in day‐to‐day vocalization rates (at one hour post‐dawn) that were only weakly explained by abiotic factors (Table 3). This suggests a prominent role for behavioral ecology in understanding the patterning of bird vocalization rates. Possible predictors include the timing of territory establishment, nest‐building, egg‐laying, incubation, hatching, and fledging. The behavior of birds on neighboring territories could also be a factor (Sillett et al., 2004). Better understanding of the relationship between reproductive behavior and signaling rate can inform acoustic sampling strategies and expand interpretations of acoustic data from times and places when observer‐based sampling is constrained, such as on military ranges or during the Covid‐19 pandemic. At longer time scales, advances in the analysis and interpretation of acoustic data can expand studies of seasonal and interannual phenology, particularly to times of year when it is difficult for researchers to access sites due to environmental conditions or competing professional demands (Marra et al., 2015). Recording devices that are timed to automatically start before breeding activity begins will make it more feasible to determine when migratory birds arrive and can spare field biologists the sometimes frustrating work of sampling to determine that the season has not yet begun.

### 4.3 Intraspecific synchrony in signaling

By combining data from multiple recording units, large synchronous acoustic datasets open a frontier of opportunities for studying behavior across landscapes (Valcu & Kempenaers, 2010). While presence/absence data are ecologically informative (Wood et al., 2021), counts of individual vocalizations can provide additional insight into behavior. We employed statistical approaches from population ecology (e.g., spline correlograms) that were developed to test for correlated dynamics in abundance and are readily extended to vocalization activity (Method 5). In our study system, the daily vocalization rates of some common species rose and fell synchronously between sites separated by 15 km (Figure 5), but not necessarily more so at 200 m than at 1500 m (Figure 6). Weather only explained a modest fraction of the spatiotemporal correlations. Other hypotheses include food availability, concordant endogenous rhythms in reproduction, or neighbor effects on behavior that extend a surprisingly long way. Playback experiments could be a powerful tool for testing hypotheses drawn from behavioral ecology (Greenfield, 1988).

### 4.4 Interspecific synchrony in signaling

Our analyses of interspecific correlations (Approach 6) suggested the existence of functional groups of species based on vocalization activity. Such structure creates opportunity for testing general theory related to acoustic partitioning, phylogenetic conservation of behavior, convergent evolution, and information transfer among species (Tobias et al., 2014). In our system, the species in cluster 1 tended to become less vocal as the breeding season progressed (Black‐throated Green Warblers, Black‐throated Blue Warblers, Black and White Warblers, Ovenbirds and Blue‐headed Vireos), whereas the species in cluster 2 tended to be less vocal on days with higher ambient sound (Red‐eyed Vireos, Black‐capped Chickadees (*Poecilia atricapillus*), Red‐breasted Nuthatches, Swainson's and Hermit Thrushes (*Catharus guttatus*), and red squirrels) (Figure 7). A priori, we would not have recognized these as different functional groups within our study system. Cluster 1 included species both with multiple clutches per year (e.g., Black‐throated Blue Warblers) and with single clutches (Ovenbirds). The two vireo species were split between clusters. The ability to identify and predict membership in vocalization guilds would contribute to basic knowledge of avian ecology, inform sampling strategies, and provide traction for predicting susceptibility to anthropogenic noise or changes in leaf‐out date.

### 4.5 Synthesis and conclusions

**FIGURE 9:**
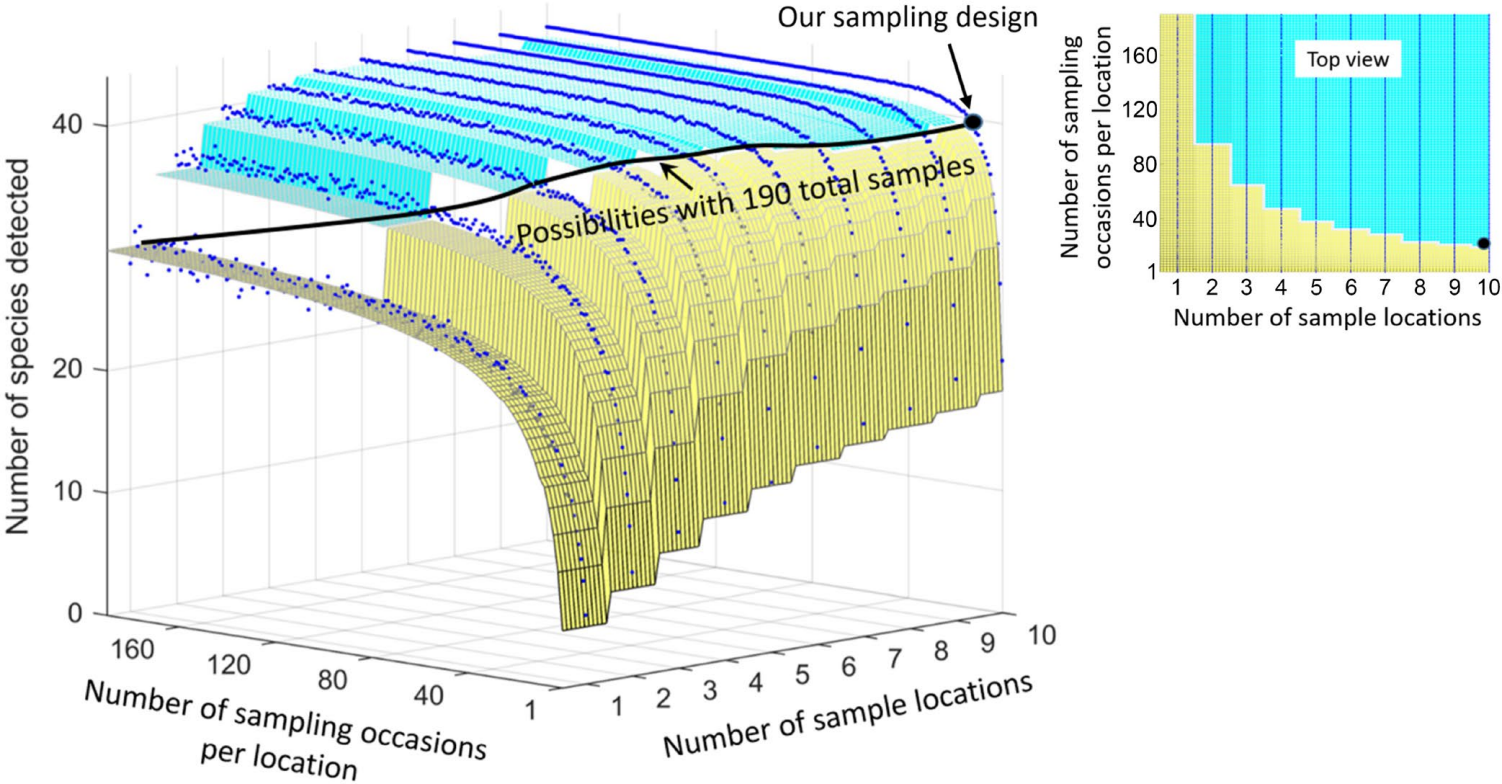
Total number of bird species detected with simulated bioacoustic sampling from different numbers of locations and sampling occasions. The boundary of the yellow and blue indicates possible combinations with analyses of 190 total sound files, as in our study; our sampling design is (10 locations × 19 occasions) is indicated at the far end of the boundary. Inset at right shows top view of same. The surface and points show averages of 1000 replicate samples drawn at random from a simulated data set modeled after our data (Appendix S1). The pattern shows that adding locations generally added to total species detections more than adding occasions

There is a natural match between data from passive acoustic monitoring and the classical concepts from community ecology of α‐, β‐, and γ‐diversity (Figure 8). These diversity metrics provide a framework for comparing communities of birds and other vocalizing animals, such as assessing how similar acoustic data from a tropical rainforest would compare with respect to α‐, β‐, and γ‐diversity to similar acoustic data from migrant songbird communities in north‐temperate forests. Such comparisons could address general ecological theory, contribute to biodiversity assessments, and contribute to optimization of sampling strategies. For example, the benefits for biodiversity assessments of adding days vs. locations from acoustic sampling will depend on the relative strength of β‐ and γ‐diversity (Figure 9). Presumably, β‐diversity will be related to the strength of seasonality and the concordance between co‐occurring species in the timing of their breeding. High γ‐diversity suggests that conservation efforts should consider relatively large management units, perhaps partly because of ecologically important variation within what appear to be uniform habitat types. The extrapolation of species accumulation curves (rarefaction) provides a tool for judging when the species composition of a community is well known vs. under‐described (Wood et al., 2021).

Passive acoustic monitoring in well‐studied sites such as the Hubbard Brook Experimental Forest allows for the synthesis and comparison of information that can be obtained from direct observations and passive recording. These comparisons will have value for studies of avian communities in other ecosystems that lack such a strong foundation from observer‐based ecological research. Having a family of technical approaches for bioacoustic data will leverage the growing power of automated data extraction, but remain critically intertwined with observer‐based field biology. Acoustic data can reveal trends, interactions, and seasonal patterns in sound pressure, but direct observations and experiments will remain crucial for asking questions, interpreting data, testing hypotheses, and developing general theory. The combination of direct observations and passive recordings offers general opportunities for understanding birds and other acoustically active organisms.

The approaches described in this paper provide tools for employing acoustic data to address basic and applied questions regarding the nature of biological communities. Passive acoustic monitoring opens sampling strategies that have historically been difficult for human observers, including collecting replicated synchronous data at many sample locations. Over time, archived recordings will continue to provide a source of data that can be mined with increasingly sophisticated detection algorithms and statistical analyses. Expanded capacity for recording and analysis is fueling growth in occupancy analysis and density estimation (Furnas & Callas, 2015; Prevost, 2016; Sebastián‐González et al., 2018), which are burgeoning fields of growing value to population ecology and conservation biology (Marques et al., 2013). Some of the approaches described here are directly related to occupancy analysis (especially approach #7, using rarefaction analysis to quantify diversity and optimize bioacoustic sampling schemes). Other approaches make use of information in vocalization rates that goes beyond presence‐absence data (especially approaches 4–6). Recent advances in recording hardware and continuing advances in data extraction software are permitting unprecedented access to acoustic data over space and time (Kahl et al., 2021; Shiu et al., 2020; Vickers et al., 2019). It seems likely that our ability to collect acoustic data will continue to exceed our capacity for analysis and interpretation. This places a premium on being strategic in framing questions, choosing hand annotation subsets, and designing analyses to evaluate acoustic data. The approaches presented in this paper provide some guideposts for analyzing, interpreting, and applying the influx of acoustic data.

Historically, soundscape analysis has often relied on assessing statistical signatures in data to understand ecological dynamics and patterns in biodiversity (Gottesman et al., 2020; Pieretti et al., 2011; Sueur et al., 2008, 2014). Incorporating detailed information about species composition and signaling rate will inform our interpretation of the patterns seen in ecoacoustic data and will enhance our ability to understanding how the acoustic signatures of environments relate to the underlying biological and ecological dynamics.

## CONFLICT OF INTEREST

The authors declare that there is no conflict of interest.

## AUTHOR CONTRIBUTIONS

**Laurel B. Symes:** Conceptualization (equal); Data curation (supporting); Formal analysis (equal); Investigation (equal); Methodology (equal); Project administration (equal); Visualization (equal); Writing – original draft (lead). **Kyle D. Kittelberger:** Conceptualization (supporting); Investigation (equal); Methodology (supporting); Validation (equal); Writing – review & editing (equal). **Sophia M. Stone:** Investigation (supporting); Validation (equal); Writing – review & editing (supporting). **Richard T. Holmes:** Validation (supporting); Writing – review & editing (equal). **Jessica S. Jones:** Data curation (equal); Methodology (supporting). **Itzel P. Castaneda Ruvalcaba:** Formal analysis (supporting); Investigation (supporting); Visualization (supporting). **Michael S. Webster:** Investigation (supporting); Project administration (supporting); Writing – review & editing (supporting). **Matthew P. Ayres:** Conceptualization (equal); Data curation (lead); Formal analysis (equal); Methodology (equal); Project administration (equal); Visualization (lead); Writing – review & editing (lead).

## Supporting information

Appendix S1

Appendix S2

## Data Availability

Recordings and annotations are accessible through the Environmental Data Initiative Data Portal: https://doi.org/10.6073/pasta/efba421dc87e077ee28010069dac7c0d.
