# Supplementary material for: Analytical approaches for evaluating passive acoustic monitoring data: A case study of avian vocalizations
Source: Ecol Evol. 2022 Apr 21;12(4):e8797. doi: 10.1002/ece3.8797 (PMC9022445; doi:10.1002/ece3.8797)
Supplement: Supplementary file 1 — Appendix S1 [file ECE3-12-e8797-s001.rtf]

Symes et al.: Approaches for analysis of avian vocalizations. Appendix S1.

Animations of georeferenced vocalization rates of 12 species at 9 locations across 21 dates from 13 May to 10 July 2018 within the Hubbard Brook watershed of the White Mountains National Forest. Symbol sizes for each location on each date are proportional to the square-root of vocalizations / 10 minute by the focal species.

Within the associated zip file, there is a gif file for each species below (sorted in order of total vocalizations recorded).

REVI_2018.gif		Red-eyed Vireo (REVI)
BTBW_2018.gif		Black-throated Blue Warbler (BTBW)
BTNW_2018.gif		Black-throated Green Warbler (BTNW)
OVEN_2018.gif		Ovenbird (OVEN)
RBNU_2018.gif		Red-breasted Nuthatch (RBNU)
BHVI_2018.gif		Blue-headed Vireo (BHVI)
HETH_2018.gif		Hermit Thrush (HETH)
SWTH_2018.gif		Swainson's Thrush (SWTH)
RESQ_2018.gif		Red Squirrel (RESQ)
YWRA_2018.gif		Yellow-rumped Warbler (YRWA)
BAWW_2018.gif	Black-and-white Warbler (BWWA)
BCCH_2018.gif		Black-capped Chickadee (BCCH)
