# Supplementary material for: Analytical approaches for evaluating passive acoustic monitoring data: A case study of avian vocalizations
Source: Ecol Evol. 2022 Apr 21;12(4):e8797. doi: 10.1002/ece3.8797 (PMC9022445; doi:10.1002/ece3.8797)
Supplement: Supplementary file 2 — Appendix S2 [file ECE3-12-e8797-s002.docx]

Supplemental Material for Symes et al., “Conceptual and analytical approaches for evaluating passive acoustic monitoring data: A case study of avian vocalizations”.

**Fig. S1.** Spectrograms of 10-min recordings with **dripping sounds manually scored as 1 on a scale of 0 – 4** (from no dripping sound to heavy sustained rain). X-axis is time (0 – 600 sec). Y axis is frequency (0 – 8 kHz). Color indicates sound amplitude at the corresponding frequency and time (low to high = black to blue to red to yellow to white). Visualized in Raven Pro 1.5 with color setting = gamma standard.


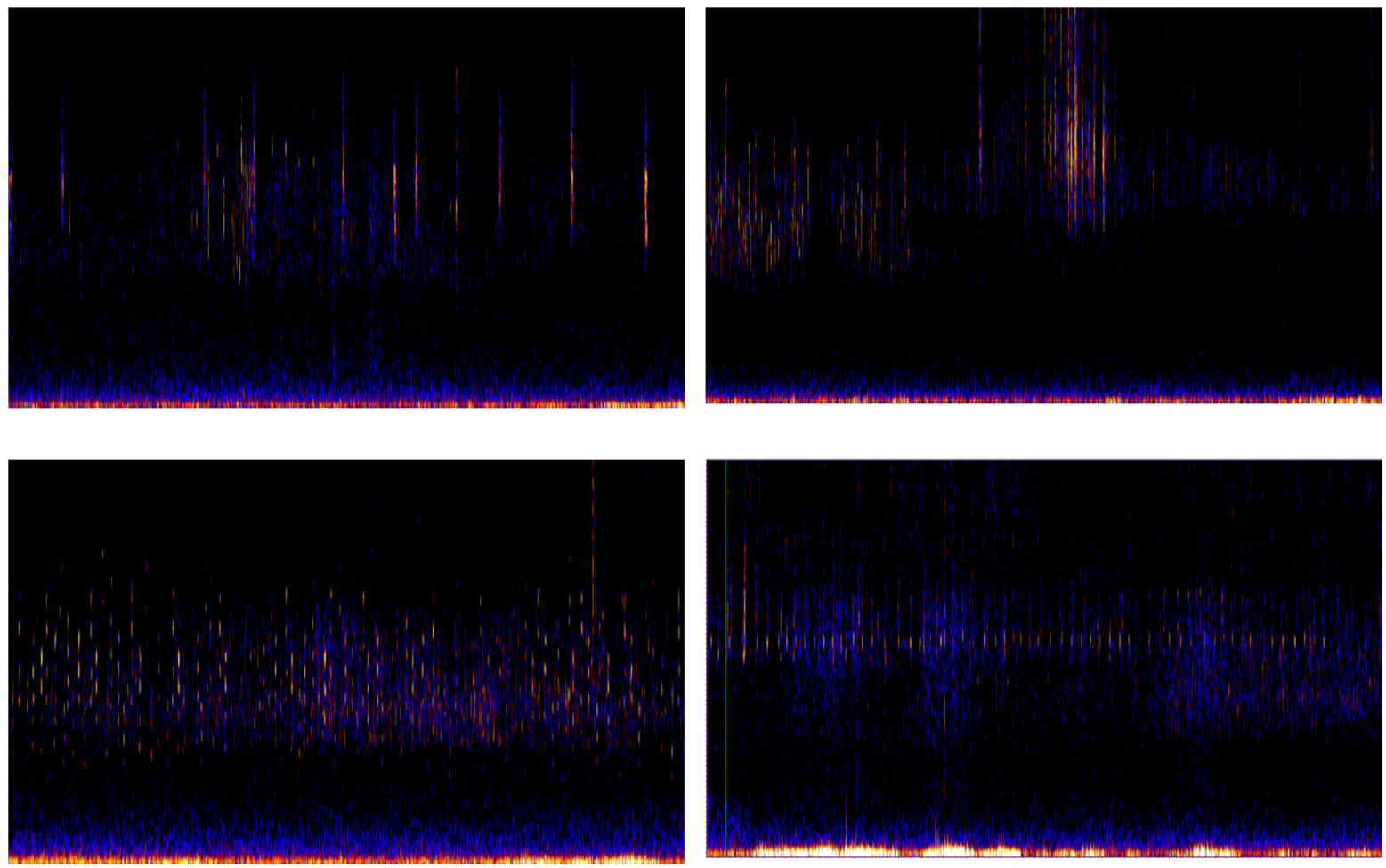


**Fig. S1, continued**. Spectrograms of 10-min recordings with **dripping sounds manually scored as 2 on a scale of 0 – 4** (from no dripping sound to heavy sustained rain). X-axis is time (0 – 600 sec). Y axis is frequency (0 – 8 kHz). Color indicates sound amplitude at the corresponding frequency and time (low to high = black to blue to red to yellow to white). Visualized in Raven Pro 1.5 with color setting = gamma standard.


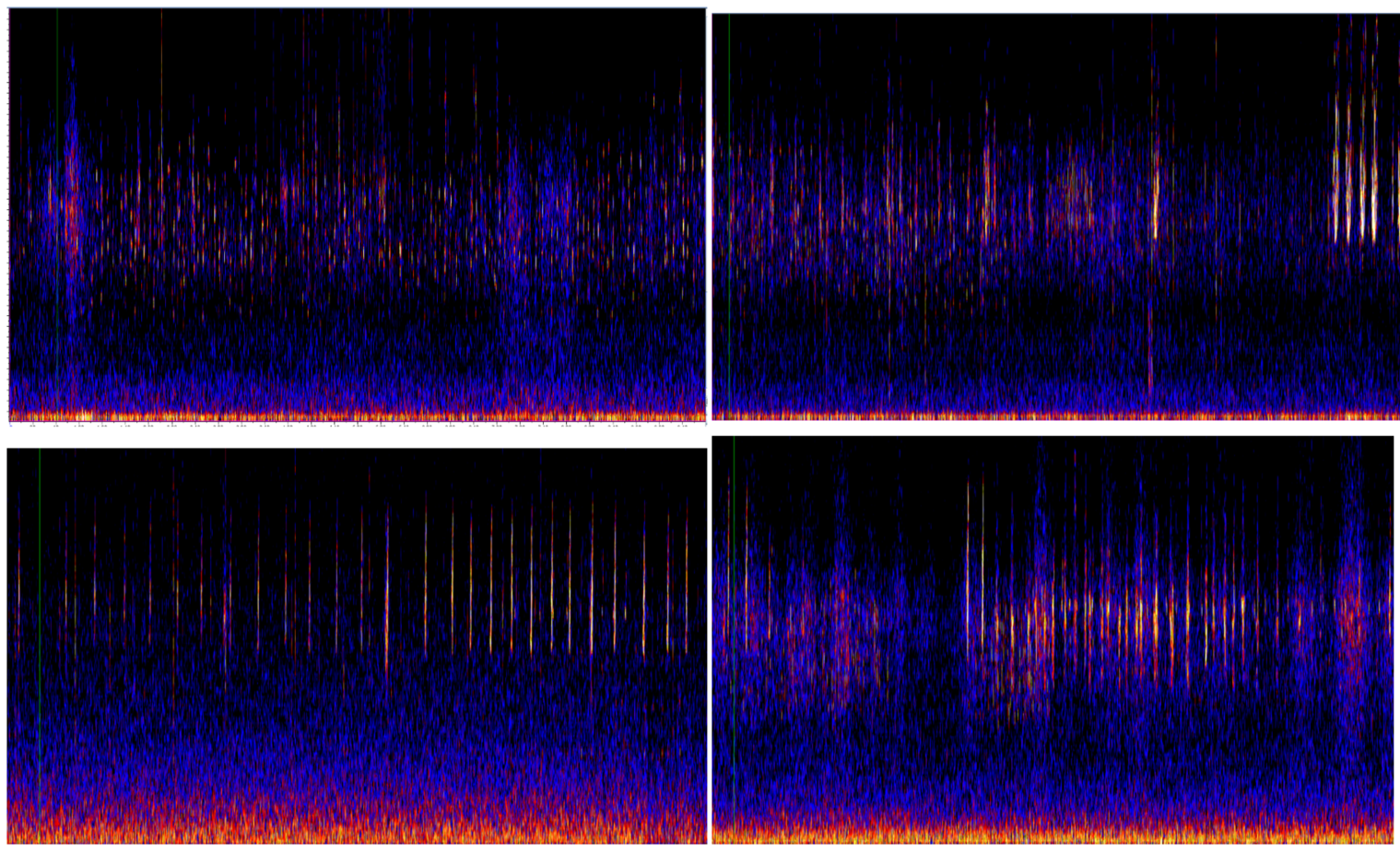


**Fig. S1, continued.** Spectrograms of 10-min recordings with **dripping sounds manually scored as 3 on a scale of 0 – 4** (from no dripping sound to heavy sustained rain). X-axis is time (0 – 600 sec). Y axis is frequency (0 – 8 kHz). Color indicates sound amplitude at the corresponding frequency and time (low to high = black to blue to red to yellow to white). Visualized in Raven Pro 1.5 with color setting = gamma standard.


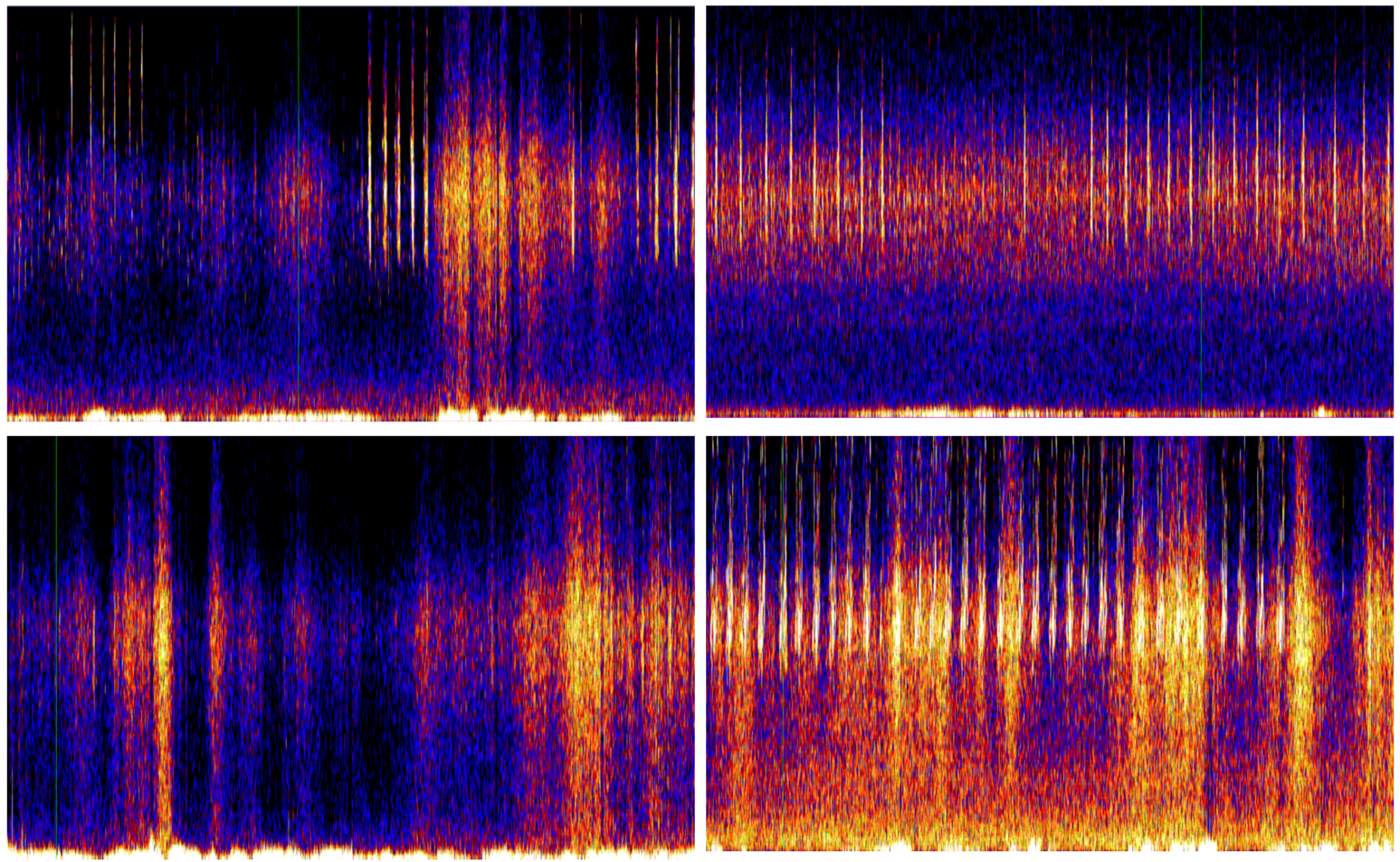


**Fig. S1, continued.** Spectrograms of 10-min recordings with **dripping sounds manually scored as 4 on a scale of 0 – 4** (from no dripping sound to heavy sustained rain). X-axis is time (0 – 600 sec). Y axis is frequency (0 – 8 kHz). Color indicates sound amplitude at the corresponding frequency and time (low to high = black to blue to red to yellow to white). Visualized in Raven Pro 1.5 with color setting = gamma standard.


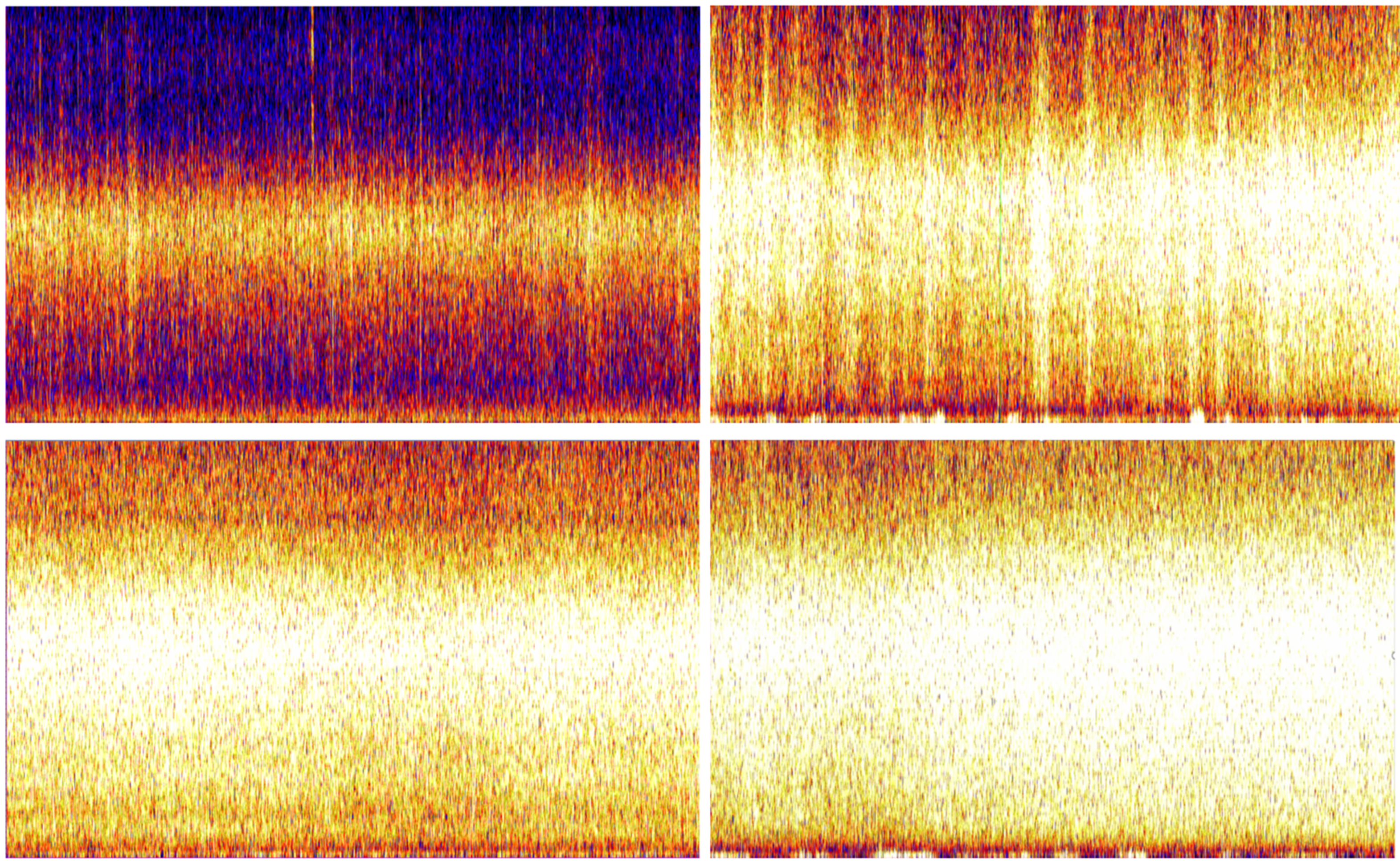


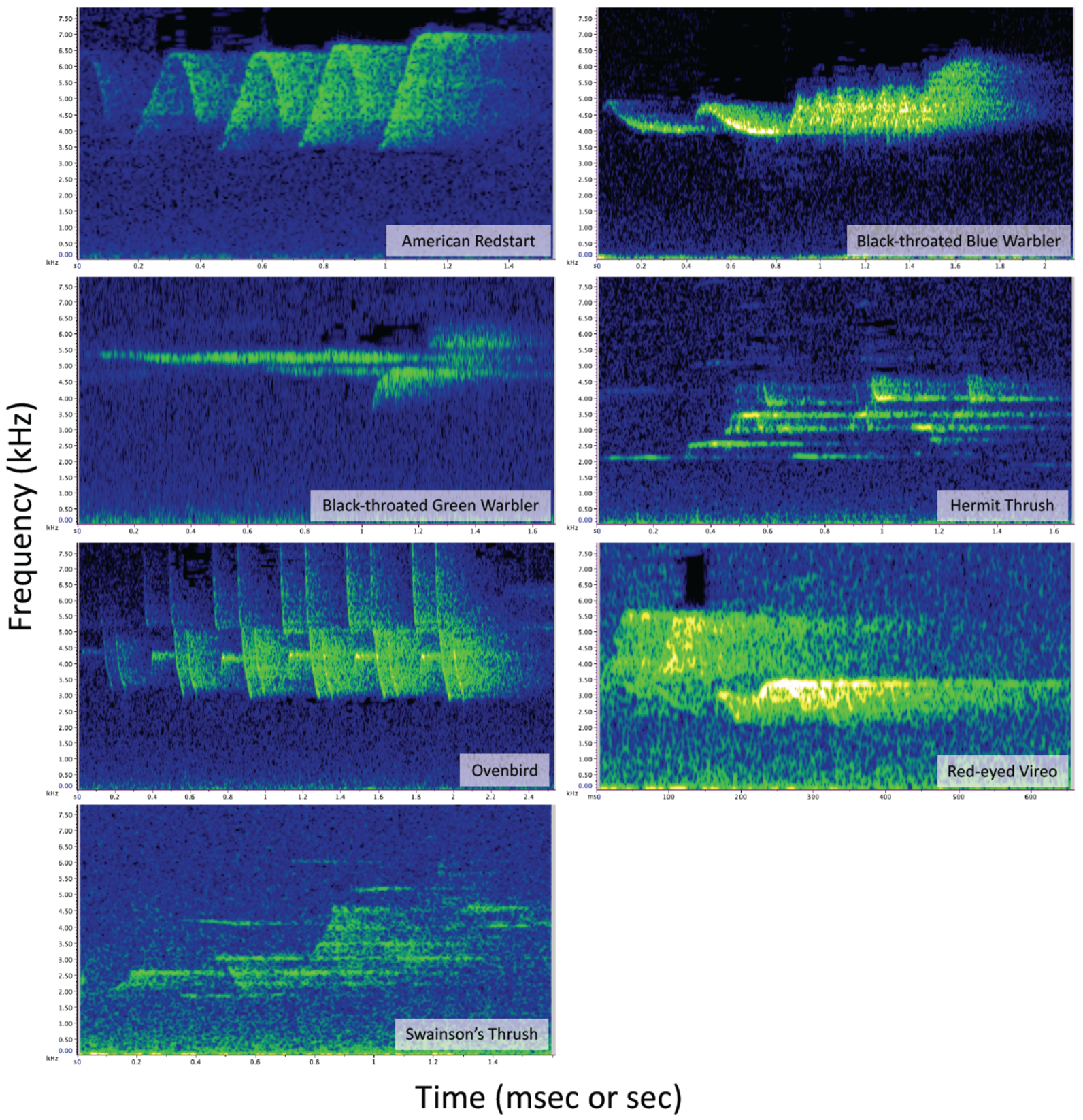


**Figure S2.** Spectrograms of typical breeding season vocalizations from seven common bird species of the White Mountains, NH. Note that the X-axes are scaled differently among species. Units of X-axis are seconds except for Red-eyed Vireo, which is in msec.


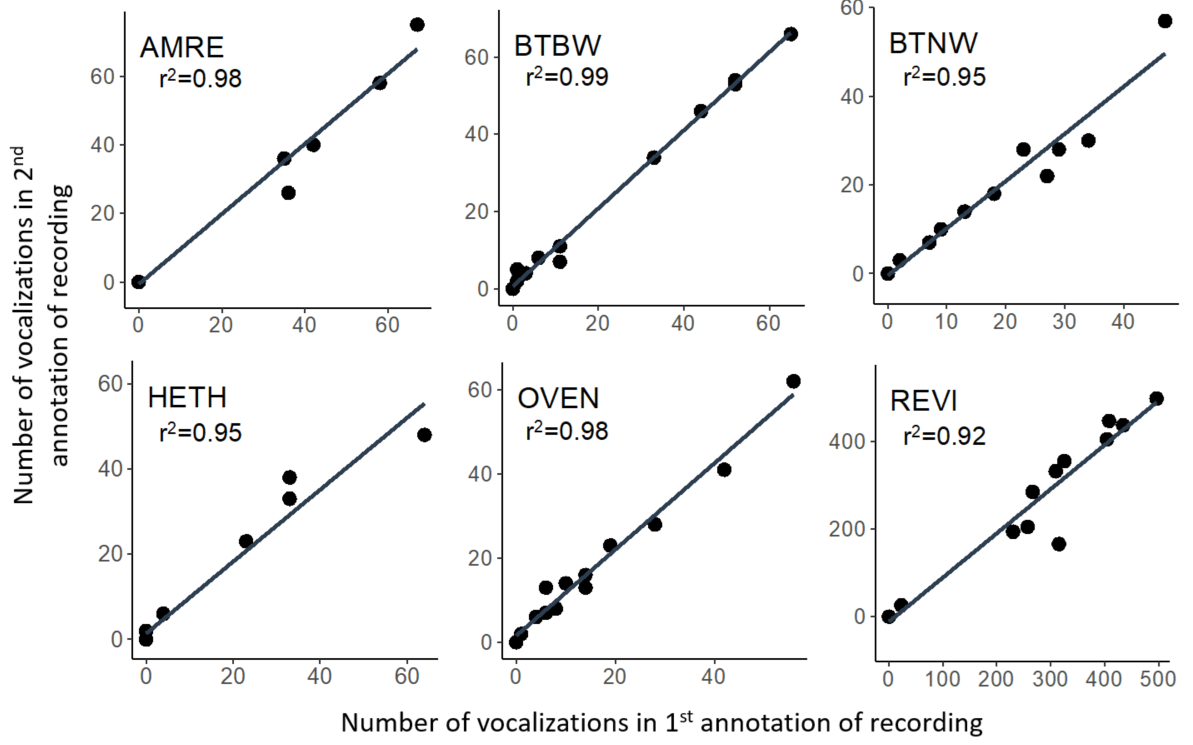


**Figure S3.** Repeatability of manual annotations from the same 13 sound files for six common bird species. The second annotations were done at a different time and without knowledge of the first annotations.


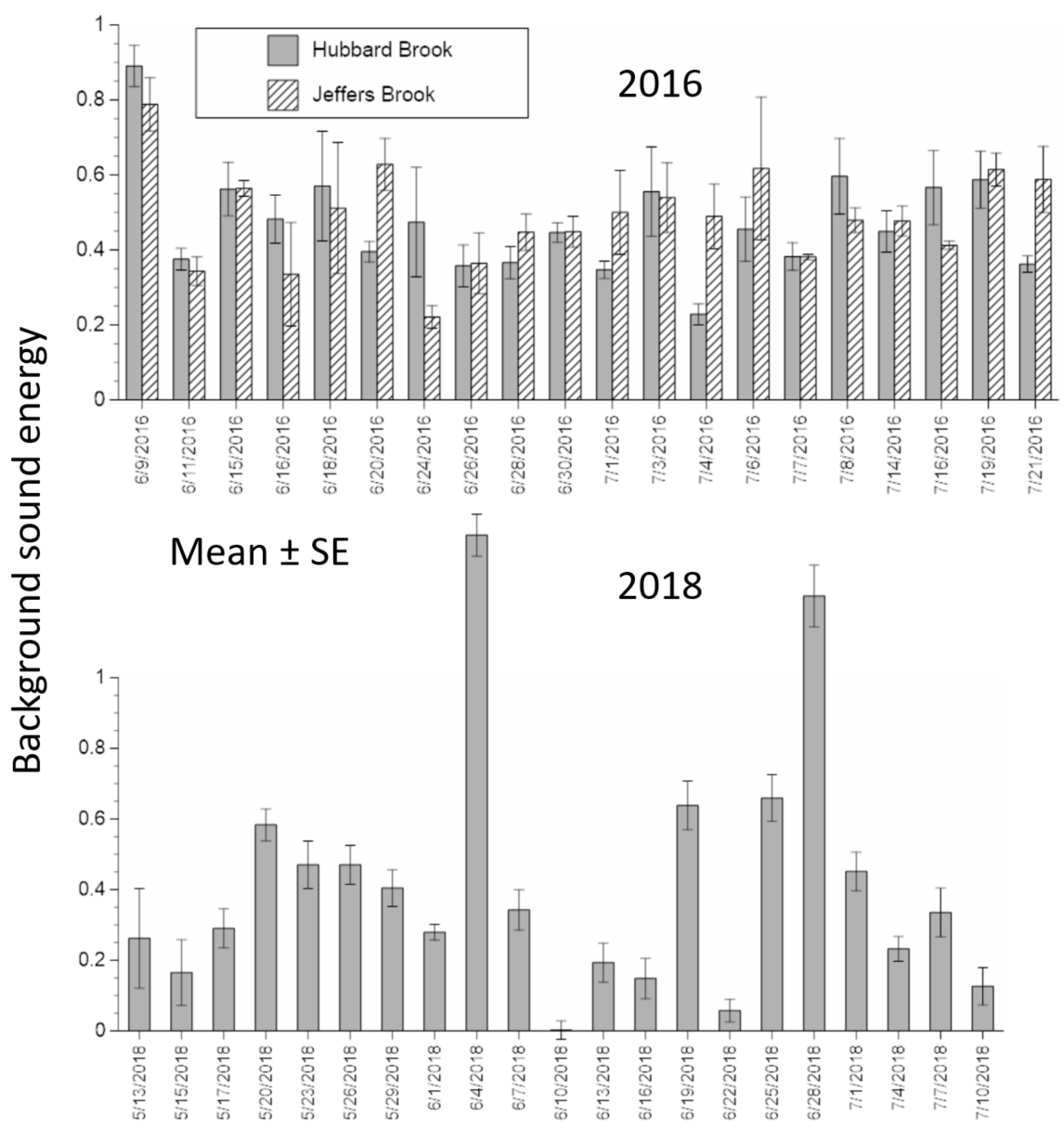


**Figure S4.** Sound energy in a typical quiet second within a 10-minute recording relative to a quiet second on a quiet day. Two days in 2018 with heavy rain had 16 – 28-fold the background sound energy of a quiet day.

**Table S1**. Attributes of recorder locations deployed at Hubbard Brook and Jeffers Brook, NH in 2016 and 2018.


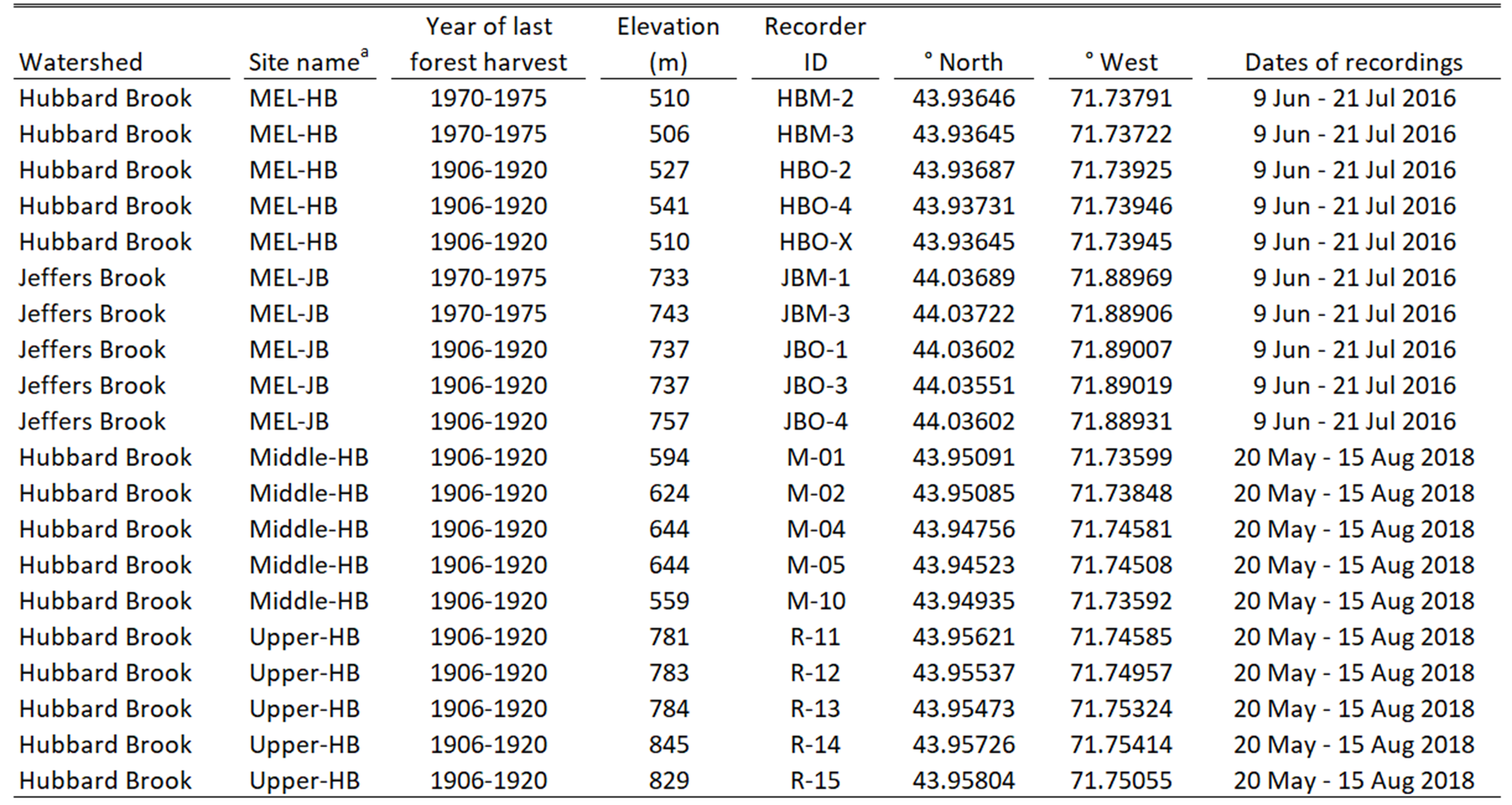


^a^ MEL = Multiple Element Limitation studies (e.g., Goswami et al. 2018. Middle and Upper = site of long term bird and insect studies (e.g., Kaiser et al. 2017, Harris et al. 2019).

**Table S2, part 1.** Summary of data types, extraction approach, and sample sizes for each of 7 technical approaches to the analysis of bioacoustic data from passive acoustic recorders.


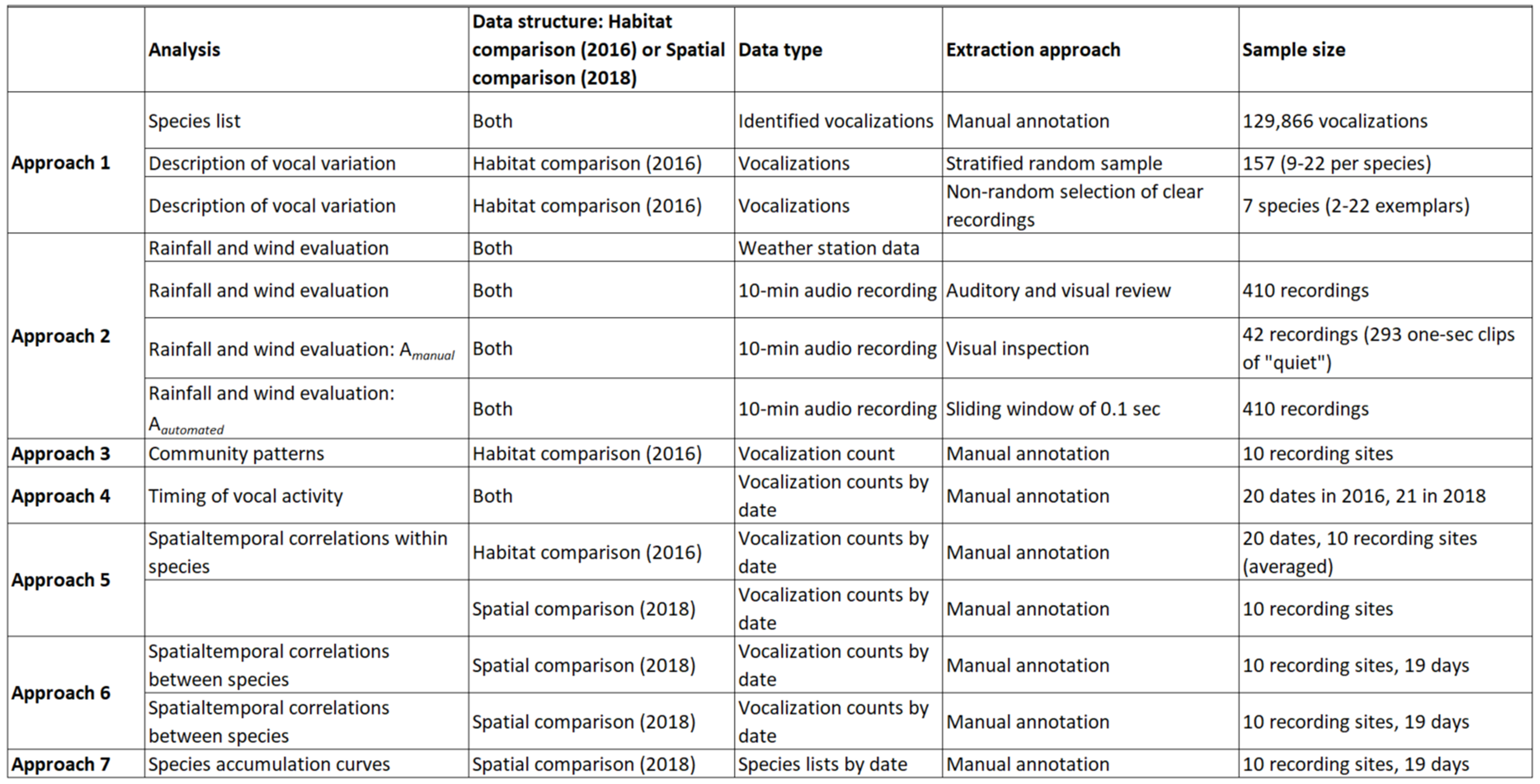


**Table S2, part 2.** Summary of analyses for each of 7 technical approaches to the analysis of bioacoustic data from passive acoustic recorders.


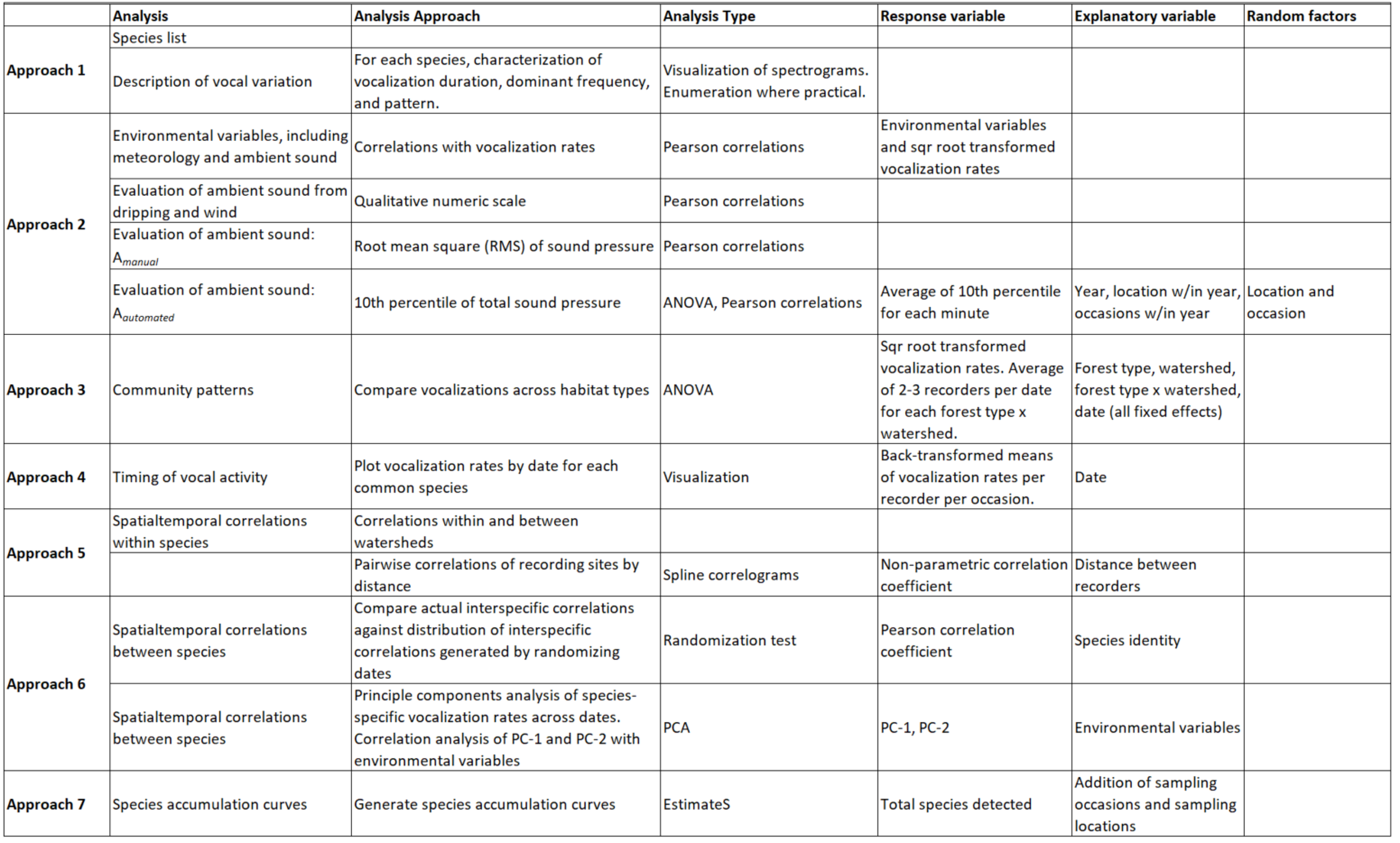


**Table S3.** For each recording, weather data, manual assessments of sound from rain and wind, and computational assessment of background sound energy.


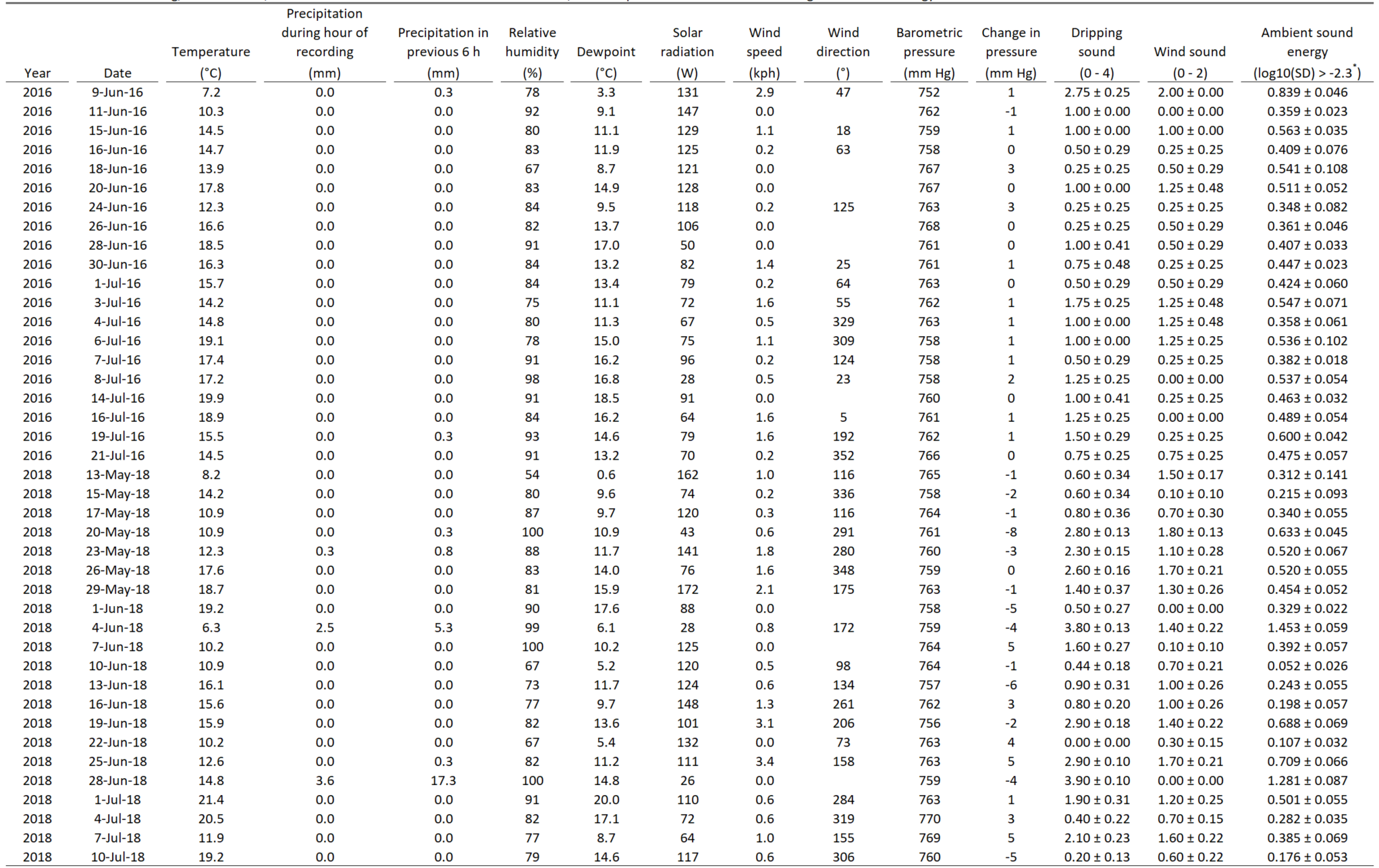


* -2.3 was the sound energy in a quiet second in our data (the 1st percentile of the distribution of sound energy in a quiet second within all 4100 minutes of our sound recordings).

**Table S4**. ANOVA results corresponding to Fig. 3. Analyses (and mean ± SD) were based on square-root transformed counts of vocalizations / 10 min.


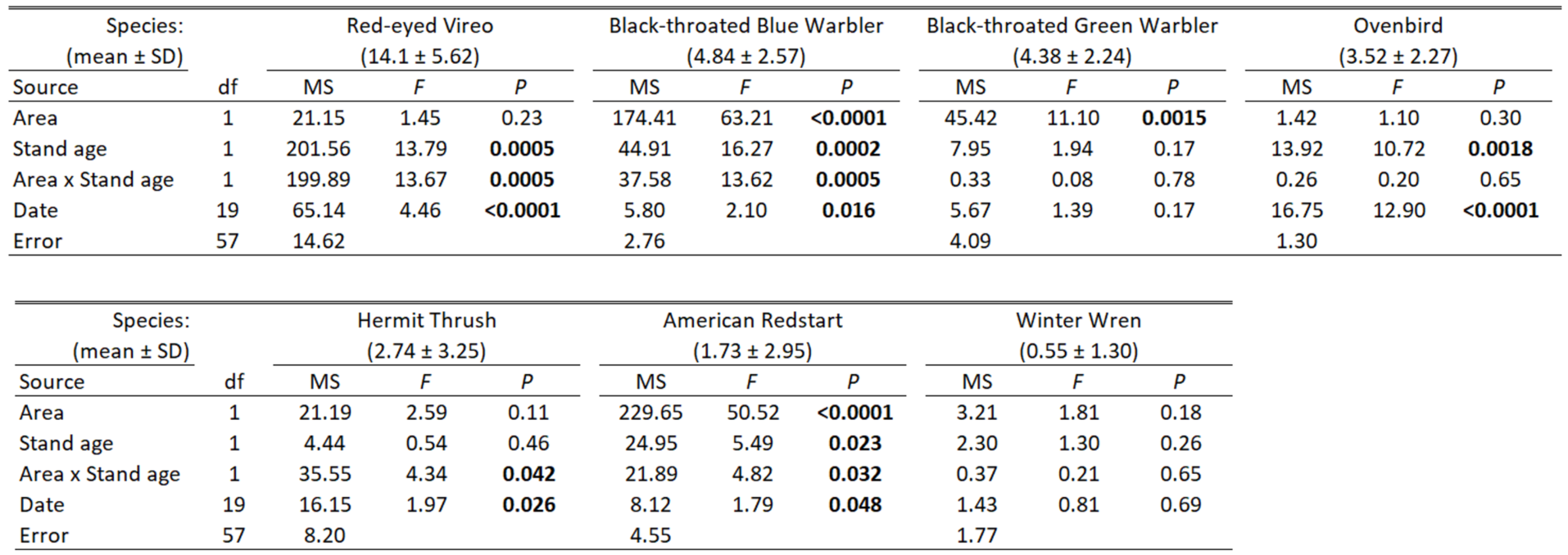


**Table S5.** Eigenvectors and loadings for principal components analysis of daily vocalization rates of 12 species across 19 dates in 2018. Corresponds to Fig. 7.


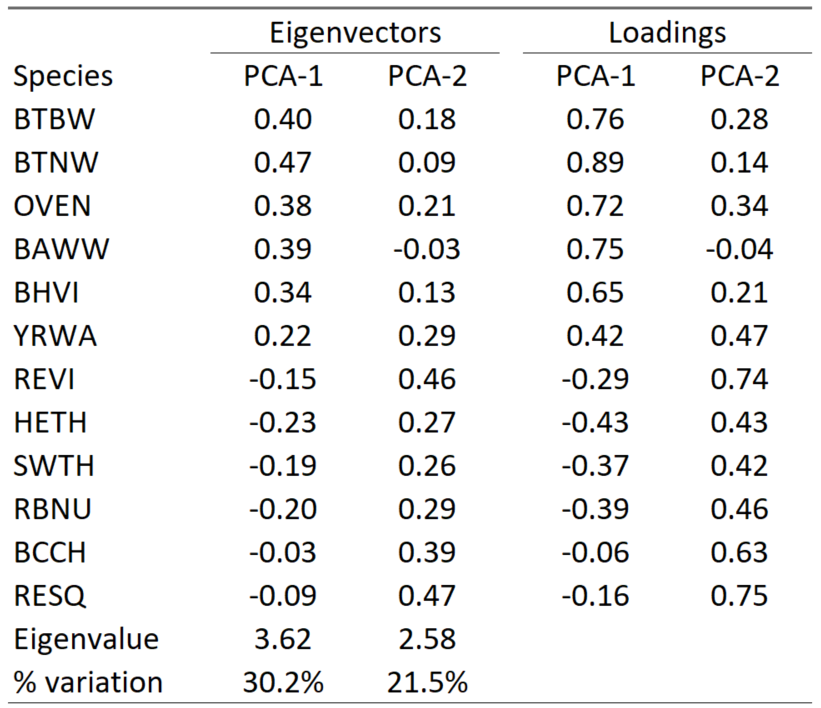


**Appendix S2.** Extrapolation of sample data for evaluating effects of varying number of recorders and sound files analyzed per recorder.

From 2018, we had annotated data for 10 recorders x 19 days. We wished to approximate the species occurrence data (presence-absence for each day) if we had been able to acquire 190 days of samples for each recorder. For each species that was detected at least once in 19 days by a recorder, we calculated the proportion of days in which it was detected. For each species *s*, *P(s)* = days detected / 19 days of sampling. Then, for each species, we simulated occurrences over 171 additional days by drawing from a random binomial distribution with probability = *P(s).*

Extrapolation of species accumulation curves for individual recorders (Fig. S4) suggested that we would have detected another 5 to 10 new species at each recorder with an additional 171 days of sampling (= *S_new_(s)* for each species *s*). To account for this, we randomly chose this number of species to be added to the simulated data at their expected occurrences. Candidate species were all those detected in our full sample, but not at the focal recorder. We chose randomly from this pool but with probabilities for each candidate species corresponding to the proportion of 10 recorders at which the species was detected. We estimated *P(s)* for each new species by extrapolation of the rank abundance curve (Figure S5) for *S_new_* new species added in the order that they were randomly drawn from the pool of candidate species. Then, for each of the new species, we simulated occurrences over 171 additional days by drawing from a random binomial distribution with probability = *P(s)*.

Theoretically, an extension of our sampling to 190 total days might have detected species that were new to the species pool after 19 days of sampling (42). However, extrapolation of our data suggested that the total pool size would not have increased with an additional 171 days of sampling (Fig S4). Therefore our simulated data of 10 recorders x 190 dates had the same global species richness (42) as in our empirical sample


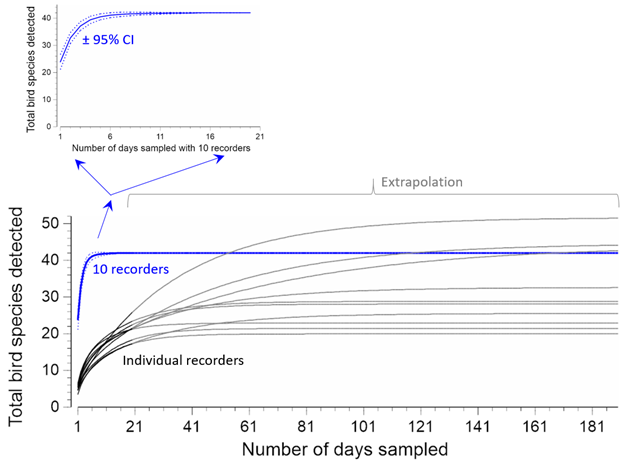


**Figure S5**. After 19 days of sampling with 10 recorders, we had detected a total of 42 species, and new species were no longer being detected (upper left). To create a larger simulated data set for comparison of different possible sampling schemes, we estimated the number of new species that would have been detected with another 171 samples from each of our recorders (below). Total possible species was restricted to ≤ 42, which affected two recorders. Analyses were performed with EstimateS 9.1.0 Biodiversity Estimation Software (Colwell 2013; S(est): equations 17 and 18 from Colwell et al. 2012 for rarefaction and extrapolation, respectively).


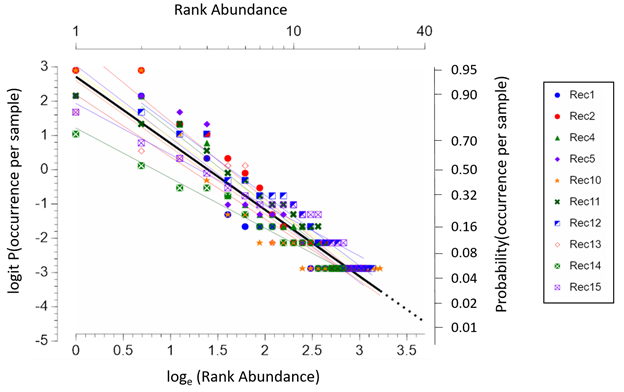


**Figure S6.** Rank abundance relations for 19 days of sampling with 10 sound recorders. The probability of occurrence per sample was the proportion of days on which each species was detected.  Species were ranked from highest to lowest proportion of occurrences. The logits of probability of occurrence were linearly related to the natural log of species rank and did not differ significantly among detectors. The overall relationship (thick black line) was logit(*Y*) = 2.71 – 1.941 · ln(*X*). The dotted dashed line indicates the region of extrapolation used for estimating occurrence per sample for hypothetical rare species expected to be detected with an increase in the number of sample days (Fig. S5).

References cited

Colwell, R. K., A. Chao, N. J. Gotelli, S.-Y. Lin, C. X. Mao, R. L. Chazdon, and J. T. Longino. 2012. Models and estimators linking individual-based and sample-based rarefaction, extrapolation, and comparison of assemblages. Journal of Plant Ecology **5**:3-21

Colwell, R. K. 2013. EstimateS: Statistical estimation of species richness and shared species from samples. Version 9. User's Guide and application published at: http://purl.oclc.org/estimates.
